# Prevention of Tungiasis and Tungiasis-Associated Morbidity Using the Plant-Based Repellent Zanzarin: A Randomized, Controlled Field Study in Rural Madagascar

**DOI:** 10.1371/journal.pntd.0002426

**Published:** 2013-09-19

**Authors:** Marlene Thielecke, Vaomalala Raharimanga, Christophe Rogier, Manuela Stauss-Grabo, Vincent Richard, Hermann Feldmeier

**Affiliations:** 1 Institute of Microbiology and Hygiene, Campus Benjamin Franklin, Charité University Medicine, Berlin, Germany; 2 Institut Pasteur de Madagascar, Antananarivo, Madagascar; 3 Faculty of Medicine, Mainz University, Mainz, Germany; University of California San Diego School of Medicine, United States of America

## Abstract

**Background:**

Tungiasis, a parasitic skin disease caused by the female sand flea *Tunga penetrans*, is a prevalent condition in impoverished communities in the tropics. In this setting, the ectoparasitosis is associated with important morbidity. It causes disfigurement and mutilation of the feet. Feasible and effective treatment is not available. So far prevention is the only means to control tungiasis-associated morbidity.

**Methodology:**

In two villages in Central Madagascar, we assessed the efficacy of the availability of closed shoes and the twice-daily application of a plant-based repellent active against sand fleas (Zanzarin) in comparison to a control group without intervention. The study population was randomized into three groups: shoe group, repellent group and control group and monitored for ten weeks. The intensity of infestation, the attack rate and the severity of tungiasis-associated morbidity were assessed every two weeks.

**Findings:**

In the repellent group, the median attack rate became zero already after two weeks. The intensity of the infestation decreased constantly during the observation period and tungiasis-associated morbidity was lowered to an insignificant level. In the shoe group, only a marginal decrease in the intensity of infestation and in the attack rate was observed. At week 10, the intensity of infestation, the attack rate and the severity score for acute tungiasis remained significantly higher in the shoe group than in the repellent group. Per protocol analysis showed that the protective effect of shoes was closely related to the regularity with which shoes were worn.

**Conclusions:**

Although shoes were requested by the villagers and wearing shoes was encouraged by the investigators at the beginning of the study, the availability of shoes only marginally influenced the attack rate of female sand fleas. The twice-daily application of a plant-based repellent active against sand fleas reduced the attack to zero and lowered tungiasis-associated morbidity to an insignificant level.

## Author Summary

Tungiasis (sand flea disease) is a parasitic skin disease present in many resource-poor communities in South America, the Caribbean and sub-Saharan Africa. In this setting tungiasis is associated with important morbidity. Hitherto, the only effective treatment is the surgical extraction of embedded sand fleas. In the endemic areas this is done using inappropriate sharp instruments and causes more harm than good. The prevention of the infestation is the only option to control morbidity. In this study we show that the twice daily application of a herbal repellent based on coconut-oil (Zanzarin), is highly effective in preventing sand flea disease in a heavily affected community in Madagascar. The attack rate became zero immediately after starting the application of the repellent. The degree of tungiasis associated morbidity approached zero within 10 weeks. In contrast, the availability of closed solid shoes had only a marginal protective effect; although shoes were requested by the villagers and wearing shoes was encouraged by the investigators at the beginning of the study. In a control group from the same village the attack rate, the intensity of infestation and of tungiasis-associated morbidity remained unchanged. Our study in rural Madagascar shows that effective und sustainable morbidity control is possible using a repellent derived from coconut oil.

## Introduction

Tungiasis is a neglected tropical disease, which is widespread in South America, the Caribbean and sub-Saharan Africa [1], [2]. It is a zoonosis with various domestic and sylvatic animals acting as reservoirs [3]. Tungiasis is associated with poverty and mainly affects marginalized populations living in urban squatter settlements, in villages in the rural hinterland or in traditional fishing communities along coastal areas [4]–[9]. Crude prevalences up to 50 percent are not uncommon in endemic areas [6]–[12]. Tungiasis is acquired when walking barefoot on soil, in which off-host stages of *T. penetrans* have propagated. 99% of all penetrated sand fleas are located at the feet [10], [13] Tungiasis may be acquired peri-domiciliary and inside houses [14].

In resource-poor settings tungiasis is associated with important and debilitating morbidity, such as intense inflammation of toes and heels, formation of painful fissures and ulcers, and deformation and loss of nails [7], [15]. Predilection sites are the toes, the heel and the sole. A common finding are walking difficulties [15], [16]. Suppuration, lymphangitis and gangrene reflect the almost constant bacterial superinfection of sand flea lesions [17]–[19]. Tetanus is a life threatening sequel of tungiasis [20], [21]. In areas where tungiasis is common and people are not or only insufficiently vaccinated against tetanus, embedded sand fleas are considered as the port of entry for *Clostridium tetani* [22].

Epidemiological studies suggest that individuals with a high parasite burden are most prone to develop severe disease sequelae [16], [23]. In endemic areas, these are children five to 14 years of age and the elderly [7], [16]. Tungiasis does not induce a protective immunity.

Hitherto, there is no effective chemotherapy available and embedded parasites need to be extracted surgically [2], [24]. This requires a skilled hand and good eyesight. In resource-poor communities, surgical removal is inconsistently performed and usually causes more harm than good, because inappropriate instruments such as pins, needles or thorns are used [16]. Since it is virtually impossible to eliminate tungiasis as long as the precarious living conditions characteristic for the endemic areas exist, and control of animal reservoirs is currently not feasible, prevention of infestation so far remains the only option.

We have previously shown that the regular application of Zanzarin, a repellent based on coconut oil, reduced the attack rate of invading sand fleas by 92%, and almost completely resolved tungiasis-associated morbidity in resource-poor settings in Northeast Brazil [25], [26]. In this study we compared the protective effect of a twice daily application of the repellent with the availability of closed shoes in a rural area in Madagascar, where the crude prevalence of tungiasis varied between 30 to 69% [6]. The decision of comparing the protective efficacy of the repellent to the protective efficacy of shoes was the result of extensive discussions with the villagers who voted for shoes as the second type of intervention when the study was planned. The decision of comparing the protective efficacy of shoes was based on extensive discussions with the villagers, who voted to receive shoes as the second type of intervention.

## Materials and Methods

### Ethics statements

**Figure 1:**
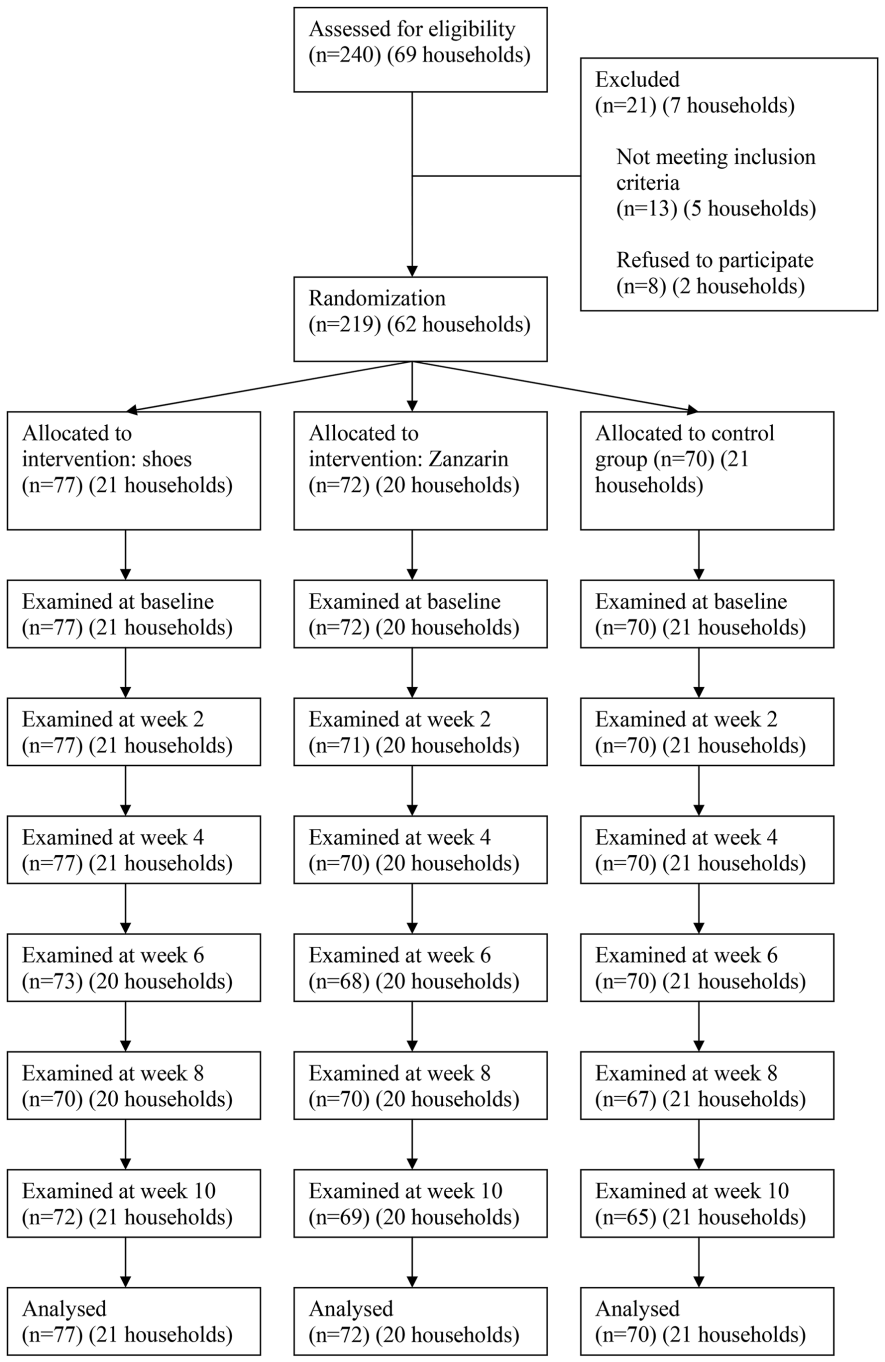
Flow diagram of the study.

The study was approved by the Ethical Committee of the Ministry of Health (MINSANP/CE ref.-nr. 051) and was registered at Controlled-trials.com (ISRCTN 11415557). The study was conducted according to the principles expressed in the Declaration of Helsinki. Informed written consent was obtained from all participants in Malagasy and in the case of minors from the parents or legal guardians. At the end of the study, all participants received a pair of closed solid shoes. In each participant, any remaining viable sand fleas were removed under sterile conditions. The flow chart of the study is depicted in Figure 1.

### Study area

The study was conducted in the villages Tanambe II (507 inhabitants) and Tanambaovao (486 inhabitants) located in Andasibe community, Moramanga district, central Madagascar. The villages are traditional communities, where people live from subsistence farming. Similar to other communities in the hinterland of Madagascar, tungiasis is common. Local people consider sand fleas disease to be inevitable and remember that it always existed.

**Figure 2:**
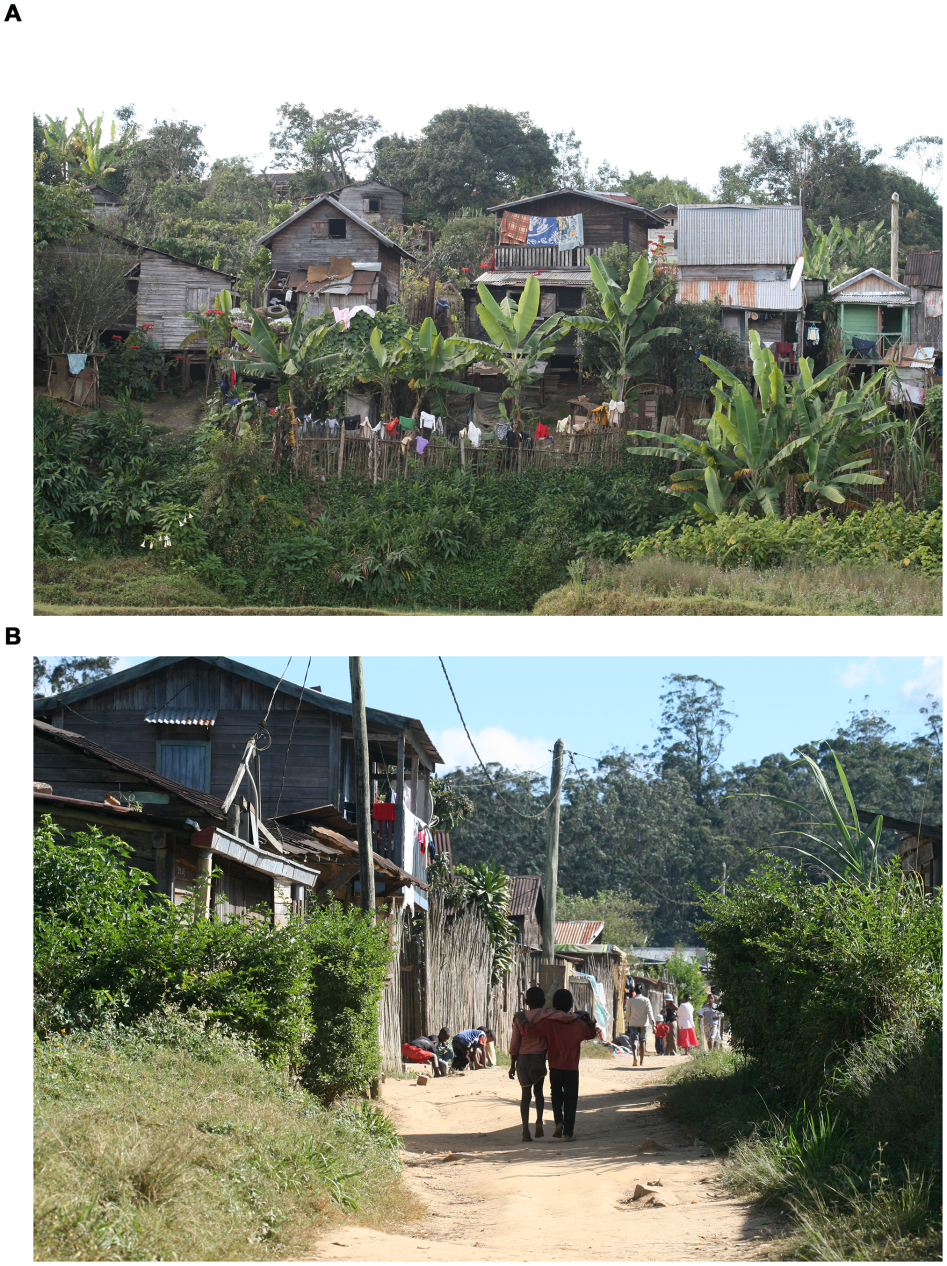
View of Tanambe II village. Houses are constructed from wood and are built on stilts (A). Roads and paths are not paved (B).

In the villages roads are not paved. The houses are made from wood and many are constructed on stilts (Figure 2A, 2B). The houses possess a front court used for cooking, washing and drying clothes. Chicken, pigs, dogs and cats are kept as domestic animals and live inside the compound. Water for cooking is derived from public pumps, water for cleaning the body and washing clothes is taken from a river close to the villages. Rice, cassava and pineapples are the main crops. Children usually do not possess shoes. Adolescents and adults have shoes, but do not wear them regularly. Most sand flea lesions are manipulated. Extraction of embedded sand fleas is attempted as soon as the lesions become painful. This is done using non-sterile instruments, such as fixing pins or needles. Neither the instrument nor the skin is disinfected. Often, the extraction is performed by elder members of the household and not by the patient himself (M. Thielecke unpublished observation 2011).

In Madagascar, the occurrence of tungiasis follows a characteristic seasonal pattern: the incidence starts to rise with the beginning of the hot and dry season (May), peaks around September and starts to decline with the beginning of the rainy season (November).

### Study design

A randomized controlled field study with three arms was carried out between May 31, 2011 (baseline investigation) and August 24, 2011 (final follow up). This period was chosen to coincide with the beginning of the dry season and the high transmission of *T. penetrans*. The study was conceived as a comparison of individuals who were provided with and encouraged to wear closed shoes and those who got a twice-daily application of the repellent to untreated individuals.

Individuals with tungiasis were identified with the assistance of community health workers. Inclusion criteria were: age ≥5 years and at least one person in the household with ≥7 lesions at both feet. The latter prerequisite was based on the rationale that, in this case, the average attack rate in household members should be high. Exclusion criteria were the presence of ≥70 lesions (total number of viable or manipulated sand flea lesions), or the presence of severe acute morbidity necessitating immediate surgical or antibiotic treatment. Eligible individuals were only enrolled if they were permanent residents in the villages, intended to stay in the community for the next four months and provided an informed written consent. In total, 240 participants were recruited and 219 were randomized.

The major outcome measures were the intensity of infestation, i.e. the number of sand flea lesions present on both feet at the time of examination, the number of newly penetrated sand fleas since the last examination (considered as a proxy of the attack rate [25], [27]), and the severity of acute and chronic pathology as measured by the severity score for acute tungiasis (SSAT) and the severity score for chronic tungiasis (SSCT) [28]. Outcome measures were assessed every two weeks by the same investigator (M. T. and V. R.). No participant was missing on more than one consecutive follow-up examination.

To avoid that members of one and the same household would participate in different treatment arms, we randomized by household. Randomization into three groups was performed using a computer-generated random number table. Participants of group I received a pair of closed solid shoes. To the feet of the participants of group II the repellent was applied twice daily. Group III did not receive any intervention. 21 households belonged to the shoe group, 20 to the repellent group and 21 households to the control group. Households with study-participants were homogenously distributed across the village. The median number of household members was 3 (interquartile range [IQR] 2–5) in the shoe, 4 (IQR 2–5) in the repellent and 3 (IQR 2–5) in the control group.

#### Shoe group

A pair of shoes was given to all members of this cohort by the research team. Before the donation of shoes, key informants of the village explained to the participants why shoes should be worn and encouraged them to wear the donated shoes regularly. They were advised not to share the shoes with other members of the household or to sell them. Shoes were closed, solid and fitted to the size of the feet. To avoid envy and mental strain all shoes were from the same type. To avoid envy and mental strain all shoes were from the same type.

Health workers who knew each participant observed the compliance of members of the shoe group by casually walking through the villages and taking notes whether a member of the shoe group wore the donated shoes or not. Compliance was assessed daily between week 2 and week 10. Individuals seen with shoes at <30% of the inspections were considered to wear protective footgear only seldom. Those who were seen with shoes at 30–60% of the occasions were considered to wear footgear irregularly. Wearing shoes at >60% of the inspections was classified as regular users.

#### Repellent group

Zanzarin (Engelhard Arzneimittel GmbH & Co. KG, Niederdorfelden, Germany) contains coconut oil (*Cocos nucifera*), jojoba oil (*Simmondsia chinesis*) and *Aloe vera*. The lotion is sold as a biocide with repellent activities against ticks and biting insects. In the morning (5:30–7:30 a.m.) and in the evening (5:00–7:00 p.m.), the repellent was applied by trained community health workers on the skin of both feet, up to the ankle including the interdigital areas, after washing the feet with water in a bowl. The average volume applied was 3 ml for 2 feet per person and day. The minimal volume was 2 ml (for children) and the maximal volume was 5 ml (for large adult feet). The procedure of applying the repellent was identical in children and adults. The application of the repellent was checked regularly by members of the team and had to be confirmed by the participant on a documentation sheet either by signing or by fingerprint. This ensured that the repellent was applied exactly as defined in the study protocol, not spilled or given away for money. The participants were asked not to wash their feet for at least two hours after the application of the repellent but they were allowed to take a shower whenever they wanted. Whether shoes already existing in a household were worn or not by the participants of the repellent group was not assessed.

#### Control group

The control group had no access to the repellent and did not receive shoes. Whether shoes already existing in the households were worn or not by the participants of the control group was not assessed.

### Definitions and documentation of lesions

The intensity of infestation, the attack rate and the degree of tungiasis-associated morbidity were assessed as described previously [25], [28]. Staging of lesions was performed according to the Fortaleza Classification [29]. The following findings were considered diagnostic for tungiasis:

- Flea in *statu penetrandi* (stage I)
- A dark and itching spot in the epidermis with a diameter of 1 to 2 mm, with or without local pain and itching (early lesion, stage II)
- Lesions presenting as a white halo with a diameter of 3 to 10 mm with a central black dot (mature egg producing flea, stage III)
- A brownish–black circular crust with or without surrounding necrosis of the epidermis (dead parasite, stage IV).

At each examination the number of viable (stage I to III) and dead (stage IV) fleas, the total number of embedded fleas ( = intensity of infestation), and the number of parasites having penetrated since the previous examination ( = attack rate) were documented. Manipulated lesions (such as partially or totally removed parasites leaving a characteristic crater-like sore in the skin), and suppurative lesions caused by the use of non-sterile perforating instruments were noted as well. At each examination data of the two feet were combined.

The exact topographic localization of each lesion, its stage, and appearance were documented on a visual record sheet and lesions were photographed, using a digital camera equipped with a macro objective (EOS 450 D, Canon, Tokyo, Japan).

Clinical pathology was assessed in a semi-quantitative manner using the severity score for acute tungiasis (SSAT), and the severity score for chronic tungiasis (SSCT) [28]. The SSAT score comprises the following signs and symptoms: erythema, edema, pain upon pressure or spontaneously, itching, sleep disturbance due to itching or pain, difficulty walking as indicated by an altered gait; abscess, and suppuration as indicators of superinfection; fissures, perilesional desquamation and ulcers as characteristic chronic skin defects. The score can take a value from 0–35 points. The SSCT ranges from 0 to 30 points and comprises the presence of nail deformation, nail loss, deformation of toes, hypertrophic nail rim, and hyperkeratosis; all of those characteristics are indicators of repeated episodes of tungiasis experienced in the past [28].

### Statistical considerations

Since the distributions of the outcome measures were skewed, the median and the interquartile ranges were used to indicate the central tendency and dispersion of data, respectively. Since the randomization was based on households, outcome measures in participants were considered to be correlated (i. e. not to be independent). To compare measurements between groups the method of generalized estimating equations (GEE) was used [30]. GEEs are appropriate to analyze longitudinal and other correlated data, especially when they are in the form of counts [30]. In all GEE models the baseline measure of the dependent variable was used as a covariate. Intention-to treat and per protocol data analyses were performed.

Assuming that the application of the repellent and the availability of shoes would have a similar protective effect, 75 participants were needed in each of the three groups to detect a difference of 50% in the intensity of infection between an intervention group and the control group (α = 5%; power of the test = 86%). A dropout rate of 25% until the end of the study was included in the calculation.

## Results

### Baseline characteristics

The demographic and clinical characteristics of the study population at baseline are shown in Table 1. There were more female participants in each group than males. This was due to the fact that many of the male inhabitants were not eligible for the study as they worked in town during the week or were absent >8 hours, so that they could not be examined. The median intensity of infestation was identical in the shoe and the control group (22 lesions), but slightly lower in the repellent group (16 lesions).

**Table 1:** Demographic and clinical characteristic of the study population.

Characteristic	Control group (n = 70)	Shoe group (n = 77)	Repellent group (n = 72)
Age in years, median (range)	25.5 (5–73)	26.7 (5–93)	26.8 (5–80)
Male-female ratio (%)	44/56	39/61	40/60
Number of household members, median (range)	3 (1–6)	3 (1–7)	4 (1–6)
Number of household members with tungiasis, median (range)	3 (1–6)	3 (1–7)	3.5 (1–6)
Intensity of infestation in household members^a^, median (range)	10.5 (2.3–33.3)	9.8 (2.3–40.3)	10.2 (1.7–27.3)
Intensity of infestation in participants^b^, median (range)	22 (1–99)	22 (1–179)	16 (1–91)
Manipulated lesions^c^, median (range)	83.2 (0–100)	88.8 (0–100)	85.4 (0–100)
SSAT^d^, median (range)	4 (0–17)	3 (0–19)	3 (0–20)
SSCT^e^, median (range)	1 (0–10)	2 (0–8.5)	2 (0–11.5)

a total number of viable, dead and manipulated lesions; only individuals with tungiasis.

b total number of viable, dead and manipulated lesions.

c the patient or a caretaker had attempted to take out the embedded sand flea; in percent of total lesions.

d severity score for acute tungiasis (see material and methods).

e severity score for chronic tungiasis (see material and methods).

### Major outcome measures

Table 2 depicts the time course of the four major outcome measures. Onward from week 2, the attack rate became zero and the intensity of infestation and the SSAT decreased significantly in the repellent group, whereas no significant change occurred in the control group. In the shoe group, there was a tendency of decrease in the intensity of infestation and of the SSAT over time.

**Table 2:** Parasitological and clinical characteristics of study groups at baseline and during intervention.

Point of time/group	Outcome measure							
	Intensity of infestation^a^	P-value^b^	Attack rate^c^	P-value^b^	SSATd)	P-value^b^	SSCT^e^	P-value^b^
**Baseline**								
Control group (n = 70)	22 (13–33)		n.a.		4 (3–7)		1 (0–2)	
Shoe group (n = 77)	22 (10–35)	0.58	n.a.		3 (2–7)	0.63	2 (0–3.5)	0.18
Repellent group (n = 72)	16 (5–31.5)	0.36	n.a.		3 (1–7)	0.50	2 (0–3)	0.15
**Week 2**								
Control group (n = 70)	21.5 (12–35)		4 (1–8)		4 (2–7)		1 (0–2)	
Shoe group (n = 77)	18 (9–34)	0.31	3 (1–6)	0.21	4 (2–6)	0.72	1 (0–3)	0.36
Repellent group (n = 71)	10 (3–27)	0.06	0 (0–1)	**< 0.001**	2 (1–4)	**< 0.001**	1 (0–2.5)	0.35
**Week 4**								
Control group (n = 70)	19 (11–32)		5 (1–9)		3 (2–7)		1 (0–2)	
Shoe group (n = 77)	17 (8–35)	0.34	3 (1–9)	0.62	3 (2–6)	0.21	1 (0–3.5)	0.52
Repellent group (70)	7.5 (2–21)	**0.02**	0 (0–1)	**< 0.001**	1 (1–3)	**< 0.001**	1 (0–2.5)	0.79
**Week 6**								
Control group (n = 70)	18 (12–36)		3.5 (1–9)		4 (2–7)		1 (0–2.5)	
Shoe group (n = 73)	17 (7–36)	0.67	2 (0–7)	0.31	3 (2–6)	0.05	1 (0.2.5)	0.32
Repellent group (n = 68)	6 (1.5–18)	**0.004**	0 (0–0)	**< 0.001**	1 (0–2)	**< 0.001**	1 (0–2.3)	0.64
**Week 8**								
Control group (n = 67)	18 (11–40)		6 (1–11)		4 (2–9)		1 (0–2.5)	
Shoe group (n = 70)	15.5 (5–41)	0.83	1.5 (0–6)	0.27	2 (1–5)	0.14	1 (0–3)	0.60
Repellent group (n = 70)	4.5 (1–17)	**< 0.002**	0 (0–1)	**< 0.001**	1 (0–2)	**< 0.001**	0.8 (0–1.5)	0.37
**Week 10**								
Control group (n = 65)	19 (10–35)		4 (2–8)		4 (2–6)		1 (0–2.5)	
Shoe group (n = 72)	15 (6–36)	0.85	2 (0–4.5)	**0.049**	3 (1–4)	0.11	1 (0–3)	0.43
Repellent group (n = 69)	3 (1–13)	**<0.001**	0 (0–0)	**<0.001**	1 (0–1)	**<0.001**	0.5 (0–2)	0.36

Data indicate the median and the interquartile range (IQR).

n. a. = not applicable.

a total number of viable, dead and manipulated lesions.

b shoe group versus control group, and repellent group versus control group, respectively.

c number of newly penetrated sand fleas since last examination.

d severity score for acute tungiasis.

e severity score for chronic tungiasis.

**Figure 3:**
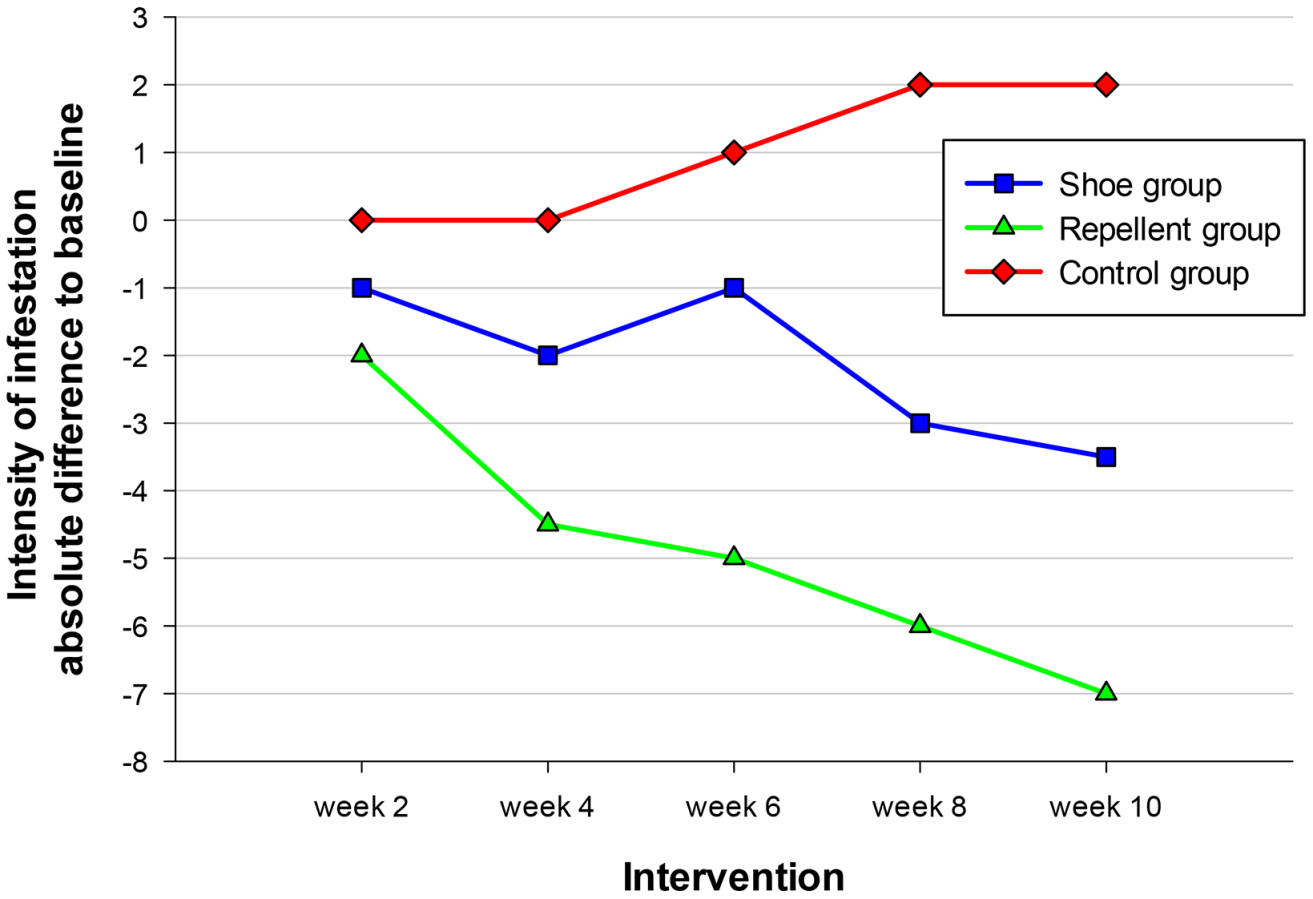
Total number of sand flea lesions (viable, dead and manipulated lesions during the intervention phase. Data indicate medians.

In order to avoid bias in the comparisons of the follow-up data between the groups, the absolute difference to baseline was calculated for the intensity of infestation, the SSAT and the SSCT. Figure 3 shows the time course of the intensity of infestation. In the repellent group the decrease was already significant at week 4 (p = 0.02 compared to the control group). In contrast, at no point of time the difference between the shoe cohort and the control cohort was significant.

**Figure 4:**
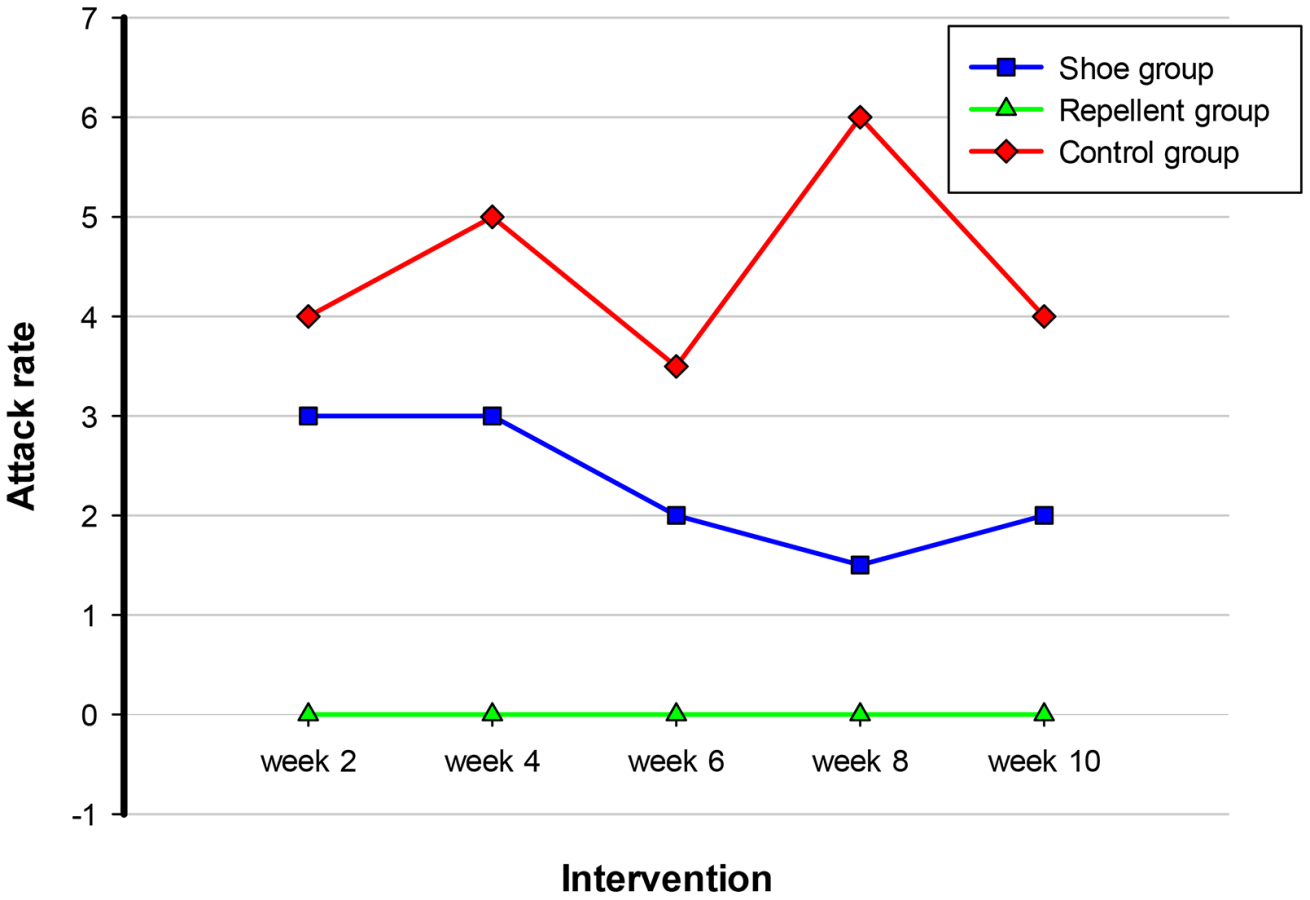
Number of newly penetrated sand fleas during the intervention phase. Data indicate medians.

The time course of the attack rate in the three cohorts is depicted in Figure 4. The median attack rate in the repellent group became zero already in week 2 and remained so until the end of the study. In contrast, in the shoe cohort the median attack rate started to decrease slightly in week 6. Only in week 10, it was significantly lower as compared to the control cohort (p = 0.049). In the control group, the attack rate varied over time.

**Figure 5:**
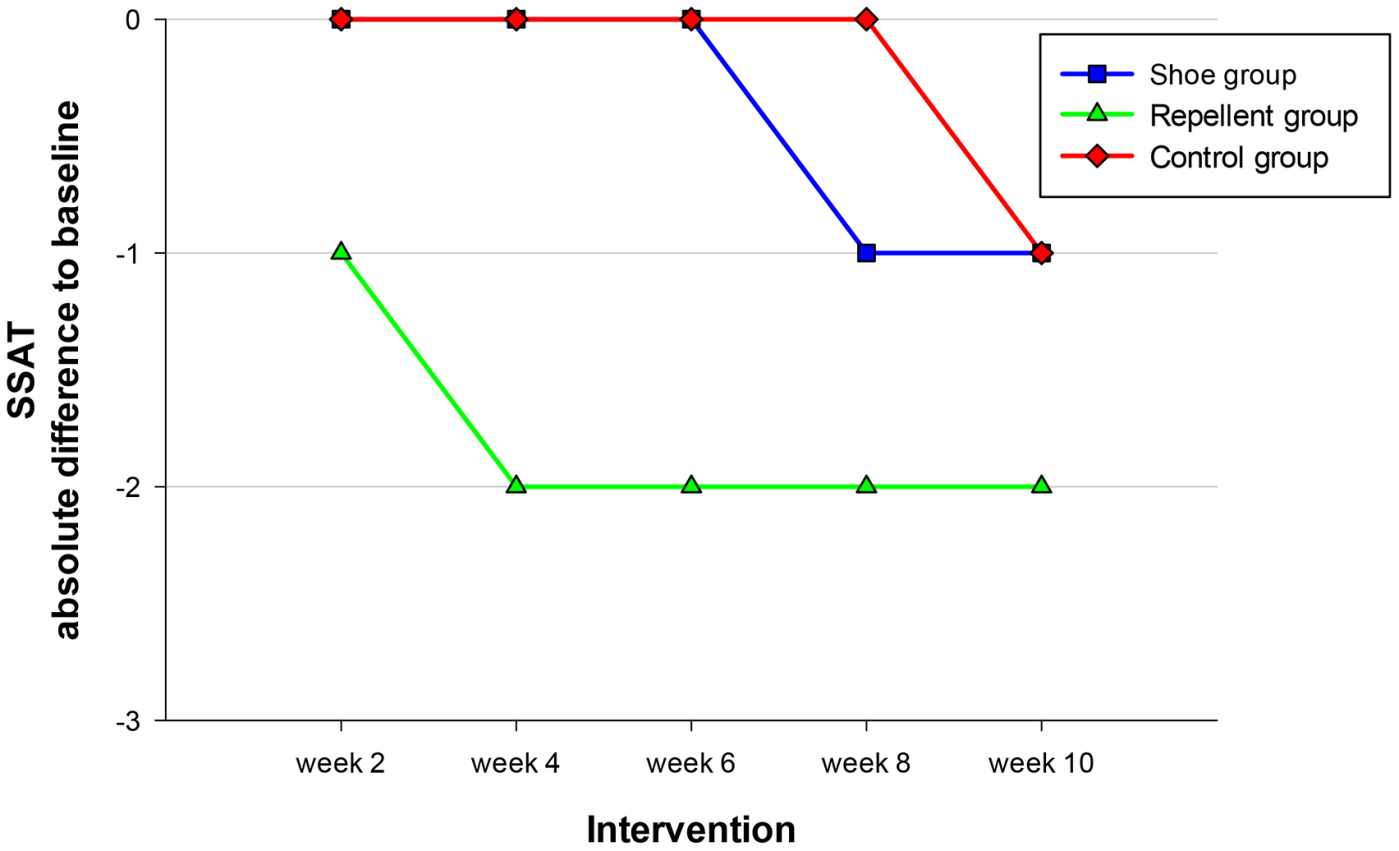
Dynamics of the Severity Score for Acute Tungiasis (SSAT) during the intervention phase. Data indicate medians.

The time course of SSAT is shown in Figure 5. At baseline, this indicator of acute tungiasis-associated morbidity was rather low in the three cohorts: median 4 (IQR 3–7), 3 (IQR 2–7) and 3 (IQR 1–7) in the control, the shoe and the repellent cohort, respectively (Table 2). Whereas in the repellent cohort the absolute difference to the baseline value became already significant in week 2 (p<0.001 compared to the control group), in the shoe cohort the absolute difference of the SSAT to the baseline value remained insignificant during all follow ups.

In all cohorts, the SSCT for chronic tungiasis was very low: median 1 (IQR 0–2), 2 (IQR 0–3.5) and 2 (IQR 0–3) in the control, the shoe and the repellent cohort, respectively (Table 2). In the repellent and the shoe cohort, a slight reduction became obvious with time. However, these differences were not significant.

To compare the efficacy of the application of the repellent with the protective effect of the availability of closed shoes, we compared the four major outcome measures at week 10. Whereas in the repellent group the intensity of infestation had decreased to a median of 3 (IQR 1–13), in the shoe group this outcome measure remained high: median 15 (IQR 6–36; p = 0.001). The attack rate was zero in the repellent group (IQR 0-0), and 2 in the shoe group (IQR 0–4.5; p = 0.03). The SSAT showed a similar tendency: median 1 (IQR 0–1) versus median 3 (IQR 1–4; p<0.0001). No significant difference between the repellent and the shoe group existed with regard to the SSCT (p = 0.17).

### Per protocol analysis

Of the 5.042 applications of Zanzarin foreseen in the repellent group, 4.832 (96%) had been applied as foreseen in the protocol. A per protocol analysis was, therefore, not considered to be meaningful in this group. Wearing shoes in the shoe group was assessed during 62 days. Fourteen participants wore shoes never/seldomly, 24 irregularly and 25 regularly; 14 were not classified, since the number of observations was too low to permit a conclusion. The average compliance was 51.6% (range 0%–98%). Stratification of members of the shoe group into individuals who never/seldomly, irregularly, or regularly wore the donated shoes is shown in Table 3. The intensity of infestation decreased continuously in the subgroup of participants wearing the shoes regularly: median 19 (IQR 11.5–29) at baseline versus median 9 (IQR 4–20) at week 10 (p = 0.03). In the subgroup of people wearing shoes irregularly the decrease was less obvious: median at baseline 18.5 (IQR 9.5–28) versus median 11.5 (IQR 5.75–28.5) at week 10 (p = 0.72). In contrast, individuals who never or rarely wore their shoes remained infested to the same degree during the entire observation period. Similarly, the attack rate decreased in participants of the two former groups, but not in individuals who never/rarely wore shoes (Table 3).

**Table 3:** The intensity of infestation and the attack rate in the shoe group stratified according to compliance.

	Wearing shoes					
	never/rarely (n = 14)	irregularly (n = 24)	regularly (n = 25)	never/rarely (n = 14)	irregularly (n = 24)	regularly (n = 25)
Point of time	Intensity of infestation^a^			Attack rate^b^		
Baseline (n = 77)	24.5 (16–55)	18.5 (9.5–28)	19 (11.5–29)	n. a.	n. a.	n. a.
Week 2 (n = 77)	23 (15–50)	13 (7.5–26)	16.5 (8–27)	2 (0–6)	2 (1–8)	2 (1–5.5)
Week 4 (n = 77)	23 (16–62)	12.5 (5–32)	11 (5–30)	5 (1–8)	1 (1–7)	3 (1–10)
Week 6 (n = 73)	30.5 (19.5–67)	12 (7–26)	12 (4–32)	4.5 (0–7)	3 (0–4)	1 (0–3)
Week 8 (n = 70)	32 (15–77)	10.5 (5.5–31)	9 (3–27)	5 (0–7)	2 (0–8.5)	0 (0–2)
Week 10 (n = 72)	21 (15–61)	11.5 (5.75–28.5)	9 (4–20)	2 (1–7)	3 (1–7)	0.5 (0–3)

n. a. = not applicable.

a total number of lesions.

b newly penetrated sand fleas since the previous examination.

Data indicate the median and the interquartile range (IQR).

At baseline, there was inverse relationship between the intensity of infestation and wearing of shoes later: median = 24.5 lesions (IQR 16–52) in participants who later wore their shoes seldomly, median = 18.5 (IQR 10–28) in participants who later wore their shoes irregularly and median = 16 (13–29) in participants who later wore their shoes regularly.

### Clinical pathology

**Figure 6:**
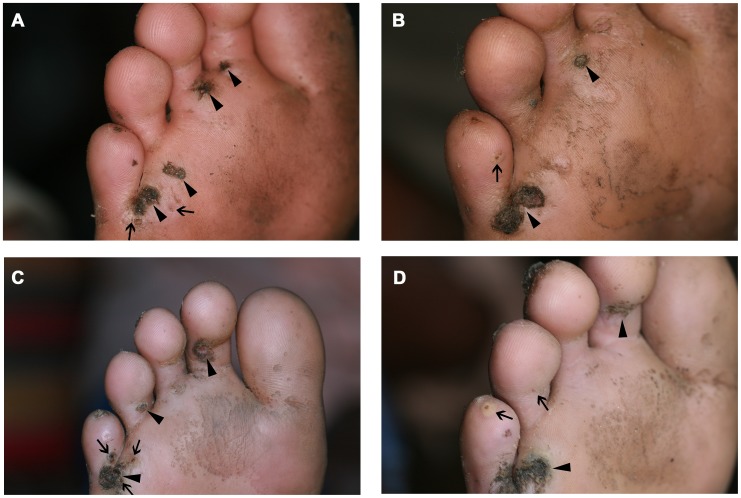
[→ triangle indicates newly penetrated sand fleas; ▴ indicates older lesions) Picture series of an individual of the control cohort with typical clinical pathology at the sole; (A) baseline examination, (B) week 2, (C) week 6 and (D) week 10 of follow up.

**Figure 7:**
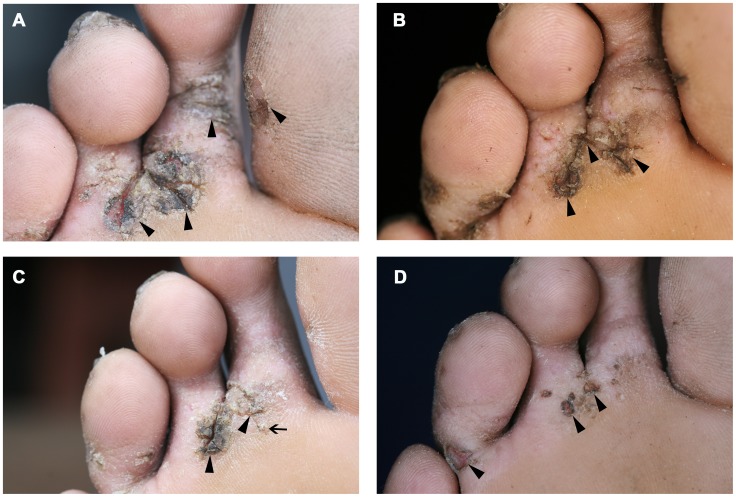
Picture series of an individual of the shoe cohort with typical clinical pathology at the sole; (A) baseline examination, (B) week 2, (C) week 6 and (D) week 10 of follow up.

**Figure 8:**
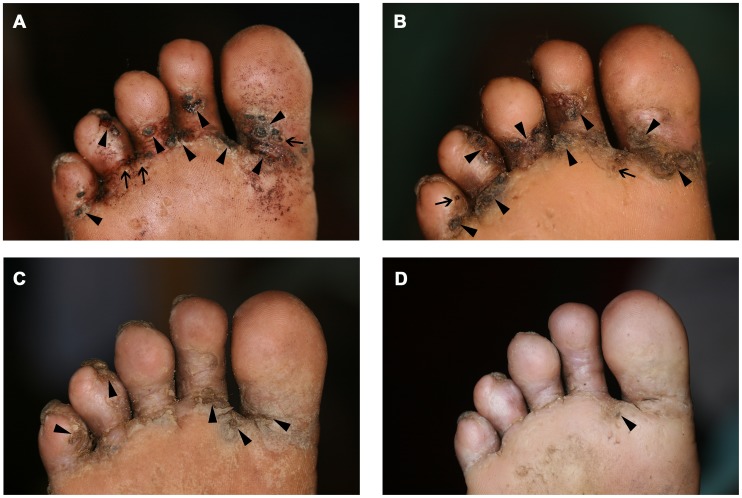
Picture series of an individual of the repellent cohort with typical clinical pathology at the sole; (A) baseline examination, (B) week 2, (C) week 6 and (D) week 10 of follow up.

Figure 6, 7 and 8 show a photo series of typical clinical pathology presentations at the soles of individuals from the control, shoe and repellent cohort. The type and degree of clinical pathology in the individual of the control cohort remained essentially the same during the observation period: several lesions disappeared and new lesions developed, and the soles remained heavily inflamed (Figure 6 A, B, C, D). In the individual of the shoe cohort, a slight amelioration was apparent onwards from week 6. However, at week 10, signs of inflammation still persisted (Figure 7 A, B, C, D). In contrast, in the individual of the repellent group, a reduction of tungiasis-related inflammation became apparent already at week 3. At week 10, the sole of this individual only showed residues of sand flea lesions without signs of inflammation (Figure 8 A, B, C, D).

## Discussion

Tungiasis is a neglected tropical disease for which no drug treatment is available [2]. During the transmission season constant reinfestation is the rule and individuals frequently harbor dozens, sometimes hundreds of embedded parasites [16]. Previous studies showed that the intensity of infestation and the degree of tungiasis-associated morbidity are correlated [16], [28]. Hence, even a partial reduction of the intensity of infestation would lower the risk for severe morbidity in an affected individual.

In the endemic areas people try to extract embedded sand fleas with kitchen utensils, sewing needles, safety pins, sharpened nails or even thorns, which usually causes more harm than good: either this induces a bacterial superinfection of the lesion or the parasite ruptures in situ, thereby, intensifying the existing inflammation [15]. Since the extraction of a burrowed sand flea with inappropriate instruments invariably causes a (micro) haemorrhage, traditional treatment may expose individuals with tungiasis at risk for transmission of viruses such as HCB and HCV in areas where the prevalence of these infections are high and non-sterile instruments are used in consecutive patients [15].

Since no drug treatment is at hand, the prevention of the infestation is the only means to control tungiasis-associated morbidity. Here we compare the efficacy of making closed shoes available by donation with the application of a repellent based on coconut oil to the feet. In previous studies in Northeast Brazil, we have demonstrated that the regular application of this repellent to the feet reversed tungiasis-associated clinical pathology to an insignificant level within four weeks [25]. Even if the repellent is only applied intermittently, e. g. daily every second week, its protective effect is remarkable [26]. In this study in rural Madagascar, the efficacy of the repellent to rapidly reduce the intensity of infestation and to resolve acute tungiasis-associated clinical pathology was also very high. This reflects the fact that the attack decreased to zero already after two weeks and remained so until the end of the study.

In tungiasis, acute clinical pathology is essentially inflammation-related, and can be measured by the SSAT [23]. Two explanations for the rapid resolution of inflammation and respectively the decrease of the SSAT in participants of the repellent group seem plausible. First, since intensity of inflammation correlates to the accumulation of penetrated sand fleas per unit of time [23], after a couple of weeks of the application of the repellent the number of remaining sand flea lesions had fallen below a threshold at which no significant acute clinical pathology develops. Second, jojoba oil and *Aloe vera*, in the concentrations present in the lotion, may have an anti-inflammatory effect and, thus, also may have contributed to the rapid decrease of inflammation-related pathology.

Within two weeks after the application of the repellent, the median attack rate was reduced to zero, and after 10 weeks even the interquartile range of this outcome measure was zero to zero. In previous studies in areas with different transmission dynamics, the twice daily application reduced the median attack rate by 92% [25], [26]. The higher efficacy observed in this study might be explained by the moderate attack rate in the two Malagasy villages in contrast to an extremely high attack rate in the Brazilian populations.

Textbooks on tropical medicine suggest that wearing footgear protects against invading sand fleas [18]. This assumption is supported by anecdotes from colonial times, indicating that in East and Central Africa local soldiers - which possessed no shoes at all - frequently developed a high intensity of infestation and severe morbidity, whereas European officers and sergeants equipped with solid footgear rarely were affected [31]. However, personal experience of two of the investigators confirms that wearing closed shoes - even with socks - does not completely protect against invading sand fleas (H. Feldmeier, unpublished observation 2004; M. Thielecke, unpublished observation 2011).

In this study, the availability of shoes had only a marginal protective effect which manifested with a delay of several weeks. At week 10, the attack rate, the intensity of infestation and the SSAT still were significantly different between the shoe group and the repellent group. Surprisingly, the compliance in the shoe group was rather low, and only 39% of group members wore the donated shoes regularly. This may have several reasons: First, in the villages wearing footwear is not a custom and people may find wearing solid closed shoes uncomfortable in comparison with flip-flops or walking barefoot. In fact, some people complained about perspiration and unpleasant odor when wearing the shoes (V. Raharimanga and M. Thielecke, unpublished observation 2011). Second, as a part of their daily routine, participants regularly came into contact with water or wet soil (e. g. when tilling their rice fields). Such work makes wearing shoes rather impracticable. Third, solid shoes are considered to be very valuable commodities in the villages, and are preferred to be used for special activities, such as going to church. Fourth, people care for their clothes very properly. Accordingly, when the donated shoes got dirty (which was almost inevitable when leaving the house), they washed and dried them. This means that shoes - even when a person was willing to wear protective footwear - were simply not available all the time. Fifth, some individuals did not wear the donated shoes in order not to be recognized as participants to avoid jealousy of other inhabitants of the village (V. Raharimanga and M. Thielecke, unpublished observation 2011). Sixth, individuals with many sand flea lesions felt pain when walking in solid shoes. This might explain why participants, never or only rarely wearing the donated shoes, were those with the highest intensity of infestation. Finally, when shoes are worn daily in rural Madagascar - where rough roads prevail and where paths are either dusty or muddy – they get cracks and are worn out after a couple of months. Frequently, they are so holey, that they should have lost any protective effect (M. Thielecke, unpublished observation 2011).

The weakness of the study is obvious. Although the wearing of shoes was encouraged at the beginning of the study, compliance was not enforced. This means, the decision whether or not to wear solid footwear was reserved for each participant itself. In contrast, the application of the repellent was performed by community health workers and strictly executed as foreseen by the protocol. Hence, no personal initiative was required from the participants of the repellent group. We deliberately decided to apply the repellent in an active way, to get in an idea to which extent tungiasis-associated morbidity can be controlled if the repellent is applied in an optimal way.

## Supporting Information

Supporting Information S1 Study protocol in French.
(DOC)

Supporting Information S2 CONSORT checklist.
(DOC)
