# Supplementary material for: Prevention of Tungiasis and Tungiasis-Associated Morbidity Using the Plant-Based Repellent Zanzarin: A Randomized, Controlled Field Study in Rural Madagascar
Source: PLoS Negl Trop Dis. 2013 Sep 19;7(9):e2426. doi: 10.1371/journal.pntd.0002426 (PMC3777867; doi:10.1371/journal.pntd.0002426)
Supplement: Supporting Information S1 — Study protocol in French. (DOC) [file pntd.0002426.s001.doc]

**DOSSIER DE PRESENTATION AU COMITE
D'ETHIQUE AUPRES DU MINISTERE DE LA SANTE
DE MADAGASCAR**

**PREVENTION DE LA MORBIDITE ASSOCIEE A
LA PUCE CHIQUE DANS UNE COMMUNAUTE A
FAIBLE RESSOURCE A MADAGASCAR**

**Financement Médecins pour le Tiers Monde (Frankfurt Allemagne)**

**Coordonnateurs :**

Docteur Vaomalala RAHARIMANGA

*Unité Epidémiologie, Institut Pasteur de Madagascar*

Marlene THIELECKE, étudiante

*Faculté de Médecine Université Charité, Berlin*

**Responsables scientifiques :**

Docteur Vincent RICHARD

*Unité Epidémiologie, Institut Pasteur de Madagascar*

Professeur Hermann FELDMEIER
*Faculté de Médecine Université Charité, Berlin*

**Co - investigateurs :**

Docteur Daniel PILGER

*Faculté de Médecine Université Charité, Berlin*

Docteur Charles Emile RAMAROKOTO

*Unité Epidémiologie, Institut Pasteur de Madagascar*

Docteur Lina RAKOTOSON

*Unité Epidémiologie, Institut Pasteur de Madagascar*

**Collaborateurs:**

Docteur Fanomezantsoa Haja Lynah RANDRIAMANANTENA
*Ministère de la Santé — Division lutte contre les Puces chiques*

**Contact :**

Docteur Vincent RICHARD

*Unité Epidémiologie Tel : (261) 20 22 401 64*

*Institut Pasteur de Madagascar Fax : (261) 20 22 415 34*

*BP 1274 - 101 Antananarivo E-mail : vrichard@pasteur.mg*

Sommaire

[RESUME DE PRESENTATION DE L'ETUDE 4](#__RefHeading___Toc351016086)

[I - INTRODUCTION 7](#__RefHeading___Toc351016087)

[1 Contexte général 7](#__RefHeading___Toc351016088)

[2 Connaissances actuelles 8](#__RefHeading___Toc351016089)

[3 Justification de l’étude 10](#__RefHeading___Toc351016090)

[II OBJECTIF 11](#__RefHeading___Toc351016091)

[III RESULTAT ATTENDU 11](#__RefHeading___Toc351016092)

[IV METHODOLOGIE 11](#__RefHeading___Toc351016093)

[1 Lieu de l’étude 11](#__RefHeading___Toc351016094)

[2 Population d’étude 12](#__RefHeading___Toc351016095)

[2.1 Critères d'inclusion 12](#__RefHeading___Toc351016096)

[2.2 Critères de non inclusion 12](#__RefHeading___Toc351016097)

[2.3 Critères d'exclusion 12](#__RefHeading___Toc351016098)

[3 Déroulement de l’étude 12](#__RefHeading___Toc351016099)

[3.1 Recensement 12](#__RefHeading___Toc351016100)

[3.2 Etude proprement dite 13](#__RefHeading___Toc351016101)

[4 Taille de l’échantillon 14](#__RefHeading___Toc351016102)

[5 Traitement des sujets à puce chique 14](#__RefHeading___Toc351016103)

[6 Recueil, gestion des données 15](#__RefHeading___Toc351016104)

[7 Analyse des résultats 15](#__RefHeading___Toc351016105)

[7.1 Description des données sociodémographiques 15](#__RefHeading___Toc351016106)

[7.2 Facteurs de risque 15](#__RefHeading___Toc351016107)

[V CONSIDERATIONS ETHIQUES 16](#__RefHeading___Toc351016108)

[1 Procédures de consentement 17](#__RefHeading___Toc351016109)

[2 Confidentialité 17](#__RefHeading___Toc351016110)

[3 Bénéfice des sujets inclus 17](#__RefHeading___Toc351016111)

[VI ARCHIVAGE DES DONNEES ET DOCUMENTS RELATIFS A L'ETUDE 18](#__RefHeading___Toc351016112)

[VII EXPLOITATION DES RESULTATS 19](#__RefHeading___Toc351016113)

[VIII FINANCEMENT 19](#__RefHeading___Toc351016114)

[IX CALENDRIER 19](#__RefHeading___Toc351016115)

[X PARTICIPANTS 20](#__RefHeading___Toc351016116)

[XI BIBLIOGRAPHIE 21](#__RefHeading___Toc351016117)

[ANNEXE 1 : Classification de Fortaleza 28](#__RefHeading___Toc351016118)

# RESUME DE PRESENTATION DE L'ETUDE

**TITRE DE L'ETUDE** : **PREVENTION DE LA MORBIDITE ASSOCIEE A LA PUCE CHIQUE DANS UNE COMMUNAUTE A FAIBLE RESSOURCE A MADAGASCAR**

**RESPONSABLES SCIENTIFIQUES**: Dr Vincent RICHARD et Prof Hermann FELDMEIER

| **OBJECTIF PRINCIPAL**  L'objectif de l'étude est de déterminer l'efficacité de Zanzarin®, un répulsif à base d'huile de noix de coco, et de la disponibilité des chaussures fermées pour réduire l'intensité de l'infestation et la morbidité associée à la puce chique chez les individus affectés par l'ectoparasite. |
| --- |

| **CONTEXTE**  Le seul traitement efficace de la puce chique incorporée est son extraction chirurgicale Celle-ci exige des matériels stériles et une bonne vision. Dans des zones endémiques, les matériels stériles ne sont pas généralement disponibles. Des aiguilles, des ciseaux, des ongles, des épines, etc. sont employés. L'extraction des puces par ces matériels non stériles cause donc plus de mal que de bien. Fréquemment, des substances toxiques sont appliquées à la peau avec l'intention de tuer les puces incorporées. A Madagascar, le badigeonnage des pieds au pétrole associe ou non à un insecticide est une mesure habituelle. Sans traitement, la puce chique évolue vers des complications souvent graves à cause de la morbidité aigüe et chronique associées. |
| --- |

| **CADRE DE L'ETUDE**  La puce chique et la morbidité associée, est un problème de santé publique négligée à Madagascar. Elle sévit dans presque toute l'île. Cependant, les prevalences changent considérablement d'une région à une autre. En milieu rural à Madagascar, la morbidité associée à la puce chique serait une menace importante pour la sante. En 2004, une étude menée chez les écoliers dans la commune de Moramanga a montré des prévalences entre 13% et 100%, avec une prévalence moyenne de 56%. |
| --- |

L'étude se déroulera en milieu rural dans 3 villages (Tanambaovao, Tanambe I, Tanambe 2), dans le fokontany d'Andasibe, district de Moramanga**.** Le choix de cette région est justifié par la prévalence de la puce chique supérieure à 40% chez les enfants pendant la saison sèche.

Une méthode rapide d'évaluation effectuée en mars 2011 a permis de mettre en évidence une prévalence estimée de 44% dans ces 3 villages.

**METHODE**

Une étude clinique contrôlée, randomisée sera réalisée. L'étude se déroulera entre mai et août 2011 dans une communauté ou la population est fortement infestée par *Tunga penetrans*. La population d'étude sera les individus plus de 5 ans, ayant au moins 1 lésion de puce chique vivante sur l'ensemble des deux pieds. La population et sera stratifiée en trois cohortes:

- une cohorte recevra le Zanzarin®, 2 fois par jour en application sur les 2 pieds.

- une cohorte recevra des chaussures solides fermées, de la taille appropriée.

- une cohorte sera le groupe témoin, et ne bénéficiera d'aucune intervention.

Les critères d'évaluation reposeront sur une série de mesures sur l'ensemble des deux pieds dont:

1. le nombre total de lésions de puces chiques (vivantes, mortes ou manipulées)
2. le nombre total de lésions de puces chiques vivantes (stade I à III de Fortaleza-Annexe 1).
3. le nombre total de lésions de puces chiques mortes (stade I qui n'évolue en stade II, stade II qui n'évolue en stade III et stade III qui n'évolue en stade I, et entre les stades il s'est écoule 8 à 10 jours).

- le taux d'attaque reflètera la dynamique locale de transmission. Le nombre de puces vivantes sera d'autant plus élevé que la transmission est importante.

- le taux de guérison reflètera l'efficacité de l'intervention. Il augmentera, si des puces chiques vivantes insérées dans la peau meurent.

- l'index de sévérité ou degré de sévérité de la morbidité aiguë et chronique associée à la puce chique se calcule par la somme des scores attribues aux manifestations cliniques liées à la puce chique (Annexe 6. Etude d'intervention de la tungose. Fiche clinique).

A la fin de l'étude toutes les puces chiques vivantes seront retirées par les personnels.

| soignants du centre de santé de base de rattachement selon le protocole en vigueur du Ministère de la Sante. La pédiculose sera aussi traite par le Nyda®. Les données seront traitées anonymement et stockées dans une base ACCESS et analysées par le logiciel EPI INFO.  **CHRONOGRAMME**  Avril 2011 : Présentation du projet au comité d'éthique,  Préparation de la mission,  Recensement, randomisation, attribution traitement  Mai - Août 2011 : Phase d'intervention  Septembre 2011 : Extraction des puces restantes  Octobre 2011 : Analyse des données  Novembre 2011- Février 2012 : Rédaction du rapport de l'étude  Fin Février 2012 : Première ébauche du manuscrit  **RESULTAT ATTENDU**  Ces données permettent au Ministère de la Santé, en particulier la Division de la Lutte contre les puces chiques, de disposer d'une stratégie de prévention efficace contre la morbidité associée aux puces chiques.  **MOTS CLES : Madagascar, puces chiques, Zanzarin®, intervention** |
| --- |

| **PREVENTION DE LA MORBIDITE ASSOCIEE A LA PUCE CHIQUE DANS UNE COMMUNAUTE A FAIBLE RESSOURCE A MADAGASCAR** |
| --- |

## I - INTRODUCTION

## 1 Contexte général

La tungose est une zoonose due à la pénétration sous la peau de l'hôte (hommes, animaux domestique ou sauvages) par la femelle adulte fécondée d'une puce appartenant à l'une des deux espèces, *Tunga penetrans* ou *Tunga trimamillata*. La puce pénètre sous l'épiderme se développe et expulse des centaines d'œufs pendant une période de 3 à 5 semaines et la majorité meurt *in situ* Les restes du parasite sont éliminées plus tard par des mécanismes de réparation de tissu (1).

A l'origine, *T. penetrans* n'était décrite que sur le continent américain. Sa première description remonte à la découverte des Amériques par les conquérants Espagnol. En 1525, la puce chique a été citée par De Oviedo y Valdes, qui a rapporté l'infestation massive par la puce chique des troupes espagnoles en Haïti (2). Deux ans plus tard, les infestations par des puces chiques ont été rapportées dans presque tous les pays du continent sud-américain que les Espagnols avaient conquis (3-5).

*T. penetrans* est une des espèces de parasite qui a migré de l'hémisphère occidental à l'hémisphère oriental. Elle a été importée en Afrique avec le sable de ballast dans un bateau naviguant du Brésil vers l'Angola en 1872. En quelques décennies, elle s'est répandue de l'Angola vers la plupart des pays de l'Afrique subsaharienne (6-8).

A Madagascar, *T. penetrans* a été mentionné pour la première fois en 1896. La puce a été apportée au pays par des marins venant du continent africain, vraisemblablement par les sénégalais d'où son appellation « parasy lafrika » ou « parasi-tsonegaly »(9). Au début, la puce chique a été circonscrite à la région nord-est de l'île et plus tard elle s'est diffusée vers toute l'île. A Antananarivo, on a identifié les premiers cas en 1899 (9).

## 2 Connaissances actuelles

La tungose s'est répandue dans les communautés urbaines et rurales à faible ressource en Afrique subsaharienne, aux Caraïbes et en Amérique du Sud, avec des prévalences entre 20 à 55% dans la population générale (10-20).

A Madagascar, elle sévit dans presque toute l'île (9). Cependant, les taux de prévalence changent considérablement d'une région à une autre. En 2004, une étude menée chez les écoliers dans la commune de Moramanga a montré des prévalences entre 13% et 100%, avec une prévalence moyenne de 56% (9). Des études récentes ont conduit à des conclusions semblables (21; 22). La présence intense de la puce chique dans une communauté se traduit par une élévation du taux d'incidence des infestations ou intensité d'infestations, c'est-à-dire le nombre de nouvelles puces (stade I) incorporées par unité de temps, et est associée à une morbidité grave (23). En milieu rural à Madagascar, la morbidité aiguë et chronique associée à la puce chique serait une menace importante pour la santé. La douleur et le prurit d'intensité différente perturbant le sommeil, les mutilations et déformations d'importance diverses, l'impotence fonctionnelle, les surinfections bactériennes et le tétanos font de cette pathologie une menace importante pour la santé de l'individu.

Des études des facteurs de risque ont prouvé que le genre masculin, la présence d'animaux domestiques dans la maison, et un logement de qualité médiocre sont des facteurs de risque de tungose. L'importance relative de chacun de ces facteurs de risque change d'une communauté à une autre (24; 25). Dans d'autres communautés, ces variables ne constituent pas des facteurs de risque. Jusqu'à présent, les études ont montré que la puce chique est une maladie associée à la pauvreté (26).

Dans les communautés rurales et urbaines à faibles ressources, l'intensité de l'infestation est habituellement élevée et les individus affectés hébergent des dizaines ou même des centaines de puces (15; 16; 27-29).

*T. penetrans* infeste une large gamme d'animaux sauvages et domestiques (30-32). Dans une étude à Fortaleza, au Brésil, respectivement 67%, 50% et 24% des chiens, chats et rats, ont été trouvés infestes (31). En milieu rural au Nigeria, les porcs sont considères comme le réservoir le plus important (25). A Madagascar, la puce chique affecte particulièrement les hommes et les porcs. En 1975, Subra et al, ont rapporté que dans la région du Lac Alaotra, *T. penetrans* était fortement liée à l'élevage de porcs (Subra *et al*, 1975, communication personnelle). La présence de *T. penetrans* sur des *Rattus rattus* et *R. norvegicus* a également été signalée à Madagascar [MALZY P.: Sur deux rongeurs importes à Madagascar In: Zoologie Malgache. ORSTOM Fonds documentaire N°20 191 Cote B.].

Selon la dynamique locale de transmission, l'infestation est acquise principalement en zone péri-domiciliaire ou á l'intérieur du domicile (33; 34). Dans des villages d'indigènes brésiliens, les stades d'évolution en dehors de l'hôte de *T. penetrans* ont été trouvés au sol, sous le lit/hamac des individus infectés, indiquant que le cycle entier de transmission pouvait être accompli à l'intérieur du domicile (34).

L'incidence change considérablement selon les saisons de l'année. Les taux d'attaque sont élevés pendant la saison sèche, mais tendent à diminuer des que la saison de pluie débute (17). A Madagascar, les taux d'attaque commencent à augmenter en mai, avec un pic de septembre à novembre et diminuent ensuite.

Les études précédentes ont montré que le taux d'incidence des infestations ou intensité d'infestation, c'est-à-dire le nombre d'individu avec des nouvelles lésions de puces chiques par unité du temps, (23; 35), peut être employé comme un indicateur pour déterminer le degré de transmission (faible ou élevé) dans un cadre de fini ou pour évaluer l'impact d'une intervention au niveau d'une communauté.

Chez l'homme, *T. penetrans* affecte en général la région peri-ungueale des orteils, des talons et de la plante du pied. Mais la pénétration peut se produire n'importe où sur le corps.

Bien que l'évolution de la tungose soit naturellement autolimitée, sans traitement elle est une maladie invalidante. Les suites de l'infestation se manifestent par des symptômes aigus de type inflammation, fissuration, ulcération, abcès, etc. et chroniques de types hyperkératoses, de formation de l'orteil et de l'ongle allant jusqu'à la perte de ce dernier. La surinfection bactérienne est très fréquente, presque systématique, et entraine des douleurs intenses (36). Les complications peuvent être des ulcères profonds, des gangrenés et des pertes d'orteil. La septicémie a été également décrite (20; 27; 37-46). On a observé le tétanos chez les individus non vaccines (18; 47-50). Les complications sont communes dans les zones où les ré-infestations sont fréquentes et où l'hygiène est défectueux (27).

## 3 Justification de l’étude

Jusqu'ici, le seul traitement efficace est l'extraction chirurgicale des puces incorporées. Elle exige des instruments stériles et une bonne vision. Dans des zones endémiques, les instruments chirurgicaux stériles ne sont généralement pas disponibles. Des aiguilles, des ciseaux, des ongles, des épines, etc. sont employés. L'extraction des puces par ces matériels non stériles peut être iatrogène (27). Fréquemment, des substances toxiques sont appliquées sur la peau dans l'intention de tuer les puces incorporées. A Madagascar, le badigeonnage des pieds avec du pétrole associe ou non à un insecticide est commun (9).

Des études récentes, dans une zone à transmission élevée, ont montré que l'application locale sur les pieds, 2 fois par jour, d'huile de noix de coco (Zanzarin®), permet de réduire le taux d'attaque, qui est le rapport entre le nombre de puces vivantes (stade I à III de la classification de Fortaleza- Annexe 1) divise par tout le nombre de lésions (stade I à V et lésion manipulée), de presque 90% (51). Si cette stratégie est poursuivie pendant la saison de transmission, elle empêche le développement de la morbidité associée à la puce chique (52).

# II OBJECTIF

L'objectif de cette étude est d'évaluer l'efficacité thérapeutique puis préventive de Zanzarin® et des chaussures fermées pour augmenter le taux de guérison et diminuer le taux d'attaque de la puce chique et en même temps réduire l'intensité de la morbidité aiguë associée chez les individus infestes par cet ectoparasite.

# III RESULTAT ATTENDU

Ces données permettent au Ministère de la Santé, en particulier la division de la lutte contre les puces chiques, de disposer d'une stratégie de prévention efficace contre les puces chiques.

# IV METHODOLOGIE

## 1 Lieu de l’étude

L'étude se déroulera dans 3 villages (Tanambaovao, Tanambe I, Tanambe 2), dans le fokontany d'Andasibe, district de Moramanga**.** Le choix de cette région est justifié par le taux de prévalence de l'infestation par la puce chique supérieur á 40% chez les enfants pendant la saison sèche (9). A l'aide d'une méthode d'évaluation rapide (62) utilisée en mars 2011 dans ces 3 villages, le taux de prévalence dans ces sites est estime à 44%.

## 2 Population d’étude

L'étude va porter sur les individus plus de 5 ans quel que soit leur sexe, qui ont au moins 1 puce chique vivante ayant pénétrées la peau sur l'ensemble des deux pieds.

### 2.1 Critères d'inclusion

- Individus âge plus de 5 ans

- Individus présents dans la zone d'étude tout au long de l'étude

- Individus avec au moins 1 lésion de puce chique vivante aux pieds

- Consentement obtenu

### 2.2 Critères de non inclusion

- Tout pour lequel le consentement n'a pas été obtenu

### 2.3 Critères d'exclusion

- Individus ayant plus de 70 lésions de puces chiques aux pieds

## 3 Déroulement de l’étude

C'est une évaluation contrôlée ouverte de l'efficacité de l'application de Zanzarin® et de la disponibilité des chaussures fermées avec examens répétés des participants. Chaque participant recevra soit le Zanzarin®, un pair de chaussures ou aucune intervention pendant une période de 10 semaines, de mai à août 2011.

### 3.1 Recensement

Un recensement de la population sera fait lors de visite à domicile et la population présente sera examinée. Pour chaque ménage, les caractéristiques démographiques, sociales, économiques, comportementaux et les coordonnées GPS du foyer seront recueillis par les médecins responsables de l'étude (Annexe 3: Etude d'intervention sur la puce chique - facteurs de risque ménage). Les facteurs de risque individuel (Annexe 2: Etude d'intervention sur la puce chique - facteurs de risque individuel) et concernant le ménage seront évalués en utilisant des questionnaires pré-testés. Ces données permettront éventuellement d'ajuster l'évaluation de l'éfficacité des interventions en fonction des facteurs de risques identifiés. Cette enquête sociodémographique a déjà obtenu l'accord du Comité National d'Ethique.

### 3.2 Etude proprement dite

L'étude proprement dite concernera les individus plus de 5 ans, ayant au moins une lésions de puce chique stade I à III (Annexe 1: Classification de Fortaleza) lors du recensement. La randomisation sera effectuée au niveau du ménage suivant trois cohortes.

Pour mesurer l'efficacité des interventions, les mesures suivantes (Annexe 6: Etude d'intervention de la tungose — Fiche clinique) seront effectuées sur chaque participant au moins 6 fois à intervalle régulier de 14 jours.

- Les critères d'évaluation reposeront sur une série de mesures sur l'ensemble des deux pieds dont:

1. le nombre total de lésions de puces chiques (vivantes, mortes ou manipulées)
2. le nombre total de lésions de puces chiques vivantes (stade I à III de Fortaleza).
3. le nombre total de lésions de puces chiques mortes (stade I qui n'évolue en stade II, stade II qui n'évolue en stade III et stade III qui n'évolue en stade I, et entre les stades il s'est écoule 10 jours)

- le taux d'attaque reflètera la dynamique locale de transmission. Le nombre de puces vivantes sera d'autant plus élevé que la transmission est importante.
- le taux de guérison reflètera l'efficacité de l'intervention. Il augmentera, si des puces chiques vivantes insérées dans la peau meurent.
- l'index de sévérité ou degré de sévérité de la morbidité aiguë et chronique associée à la puce chique se calcule par la somme des scores attribues aux manifestations cliniques liées à la puce chique (Annexe 6. Etude d'intervention de la tungose. Fiche clinique)
- les lésions caractéristiques seront photographiées avec un appareil photographique numérique de haute résolution équipe d'un macroobjectif, au début et à la fin de chaque
- période d'intervention.

A chaque visite, les démangeaisons et les douleurs seront évaluées en utilisant des échelles ordinales visuelles pour tous les participants (Annexe 5: qualité du sommeil en rapport avec prurit et douleur) (Worth et al, 2010).

A la fin de l'étude (à la fin de la troisième période d'intervention), toutes les puces chiques vivantes restantes seront extirpées au Centre de Santé de base de rattachement selon le protocole en vigueur du Ministère de la Santé. La plaie sera désinfectée et un antibiotique local sera applique.

Tous les membres du ménage infestes par les poux C*apitis pediculosis* seront traites avec Nyda®.

## 4 Taille de l’échantillon

Pour détecter une différence attendue de 50% entre la cohorte de Zanzarin® et la cohorte des chaussures (risque α de 5%, puissance du test à 86%) et en supposant un taux d'abandon de 25% au cours des trois mois de l'étude, 75 individus sont nécessaires dans chaque cohorte. Par conséquent, la population totale d'étude devra comporter 225 individus.

## 5 Traitement des sujets à puce chique

A la fin de l'étude, toutes les puces vivantes restantes seront extirpées. La blessure sera désinfectée et un antibiotique local sera appliqué. Cet acte sera confié au Centre de Santé le plus proche et pris en charge par le projet pour le déplacement et le traitement. En général, cette intervention ne nécessite pas d'hospitalisation mais doit être couverte par une vaccination antitétanique et éventuellement par une sérothérapie antitétanique dans le cas où l'enfant n'est pas à jour de sa vaccination.

## 6 Recueil, gestion des données

Les données seront saisies sur le principe de la double saisie dans une base de données en utilisant le logiciel Access 2003 et seront analysées avec le logiciel Epi Info software package (version 6.04d; Centers for Disease Control and Prevention, Atlanta, USA) pour vérifier les erreurs qui ont pu être produites pendant leur entrée.

## 7 Analyse des résultats

### 7.1 Description des données sociodémographiques

### 7.2 Facteurs de risque

Les fréquences relatives seront comparées par le test du Chi-2. Les différences entre les variables quantitatives seront évaluées par le test de Wilcoxon.

Les variables jugées significatives lors de l'analyse bivariée (ayant une valeur de p<0,2 choisit arbitrairement) seront introduites dans un modèle de régression logistique multi variée pour déterminer les facteurs de risque.

8-3- Evaluation de l'efficacité des 2 produits.

Les premières mesures sont le taux d'attaque et l´intensité d´infestation.

La deuxième mesure est l’intensité de la pathologie clinique, mesuré par le Severity Score of acute tungiasis (SSAT) et le Severity Score of chronic tungiasis (SSCT).

L'objectif des premières mesures est de mettre en évidence une activité préventive des deux interventions mise en évidence par un écart du taux d'attaque d'au moins 50% entre le témoin et les deux autres cohortes.

L'objectif de la deuxième mesure est de mettre en évidence une activité curative des deux interventions mise en évidence par un écart du taux de guérison d'au moins 50% entre le témoin et les deux autres cohortes.

Les mesures sont répétées au moins 6 fois à intervalle régulier de 14 jours.

Une analyse en intention de traiter qui consiste à analyser les résultats des patients dans leur groupe de randomisation initiale, quel que soit le traitement qu'ils aient réellement reçu et quelle que soit leur évolution par rapport à l'étude sera entreprise.

La morbidité associée à la puce chique sera déterminée en utilisant des degrés de sévérité précédemment établis pour la morbidité aigue et chronique associée (23) (Annexe 1: étude d'intervention sur la puce chique: fiche clinique).

Le critère de jugement sera l'efficacité de l'intervention. Il est reflété par une augmentation du taux de guérison et la diminution du degré de sévérité de morbidité associée à la puce chique.

# V CONSIDERATIONS ETHIQUES

Cette étude est continué conformément au protocole et aux recommandations internationales en terme d'études cliniques (déclaration d'Helsinki, adoptée par l'Assemblée Mondiale en 1964, recommandations des Bonnes Pratiques Cliniques).

Le protocole de cette étude sera soumis pour avis au Comité d'Ethique auprès du Ministère de la Santé de Madagascar. Toute modification susceptible d'intervenir par rapport au protocole actuel, concernant les modalités de l'étude et tout particulièrement la prise en charge des patients inclus, fera l'objet d'un amendement au protocole soumis au Comité d'Ethique auprès du Ministère de la Santé de Madagascar.

Le Comité d'Ethique auprès du Ministère de la Santé de Madagascar sera destinataire du rapport final de cette étude.

## 1 Procédures de consentement

Une information orale sera d'abord donnée à toutes les communautés lors d'une réunion. Elle sera donnée par le médecin responsable de la circonscription médicale, le chef de village et les médecins responsables de l'étude.

Une information orale et écrite (Annexe 8: Information du patient avant inclusion) en malgache sera encore fournie aux parents ou tuteurs de l'enfant avant l'obtention de leur consentement écrit de participer ä l'étude, expliquant les objectifs, les modalités pratiques (questionnaire, suivi), le résultat attendu, la gestion de la confidentialité des données, leur droit au refus de participer à l'étude et la possibilité de laisser à chaque sujet le choix de quitter l'étude à tout moment.

Un consentement éclaire sera signe (ou empreinte digitale éventuellement) par un des 2 parents ou un tuteur de l'enfant (Annexe 9: Formulaire de consentement)

Quand le consentement de participation à l'étude sera obtenu, un des deux parents ou le tueur et l'enfant seront interrogés et les informations seront recueillies dans un formulaire.

## 2 Confidentialité

Les personnels qui recueilleront les données propres à l'enquête sont tenus par le secret professionnel.

## 3 Bénéfice des sujets inclus

A la fin de l'étude:

- Toutes les puces vivantes restantes seront extirpées au Centre de Santé de base le plus proche selon le protocole du Ministère de la Santé et les enfants seront vaccinés contre le tétanos en cas de besoin. La blessure sera désinfectée et un antibiotique topique sera appliqué.

- Tous les membres du ménage infestés par les poux C*apitis pediculosis* seront traités avec Nyda®.

Au cours des visites de suivi à domicile, tout cas suspect de paludisme chez les enfants et les membres de sa famille bénéficieront d'un test rapide du paludisme et seront adresse au dispensaire le plus proche pour la prise en charge éventuelle s'il le souhaite.

#

# VI ARCHIVAGE DES DONNEES ET DOCUMENTS RELATIFS A L'ETUDE

Conformément aux prescriptions internationales dans ce domaine, un exemplaire de tout document relatif à l'étude ainsi que du fichier informatise de saisie de la base de données seront conservés pendant une durée de 15 ans à l’Institut Pasteur de Madagascar.

Toutes les précautions seront mises en œuvre de manière á préserver la confidentialité des données recueillies auprès des sujets. Ces précautions concernent en particulier le domaine de l'archivage des formulaires de l'enquête (local ferme à clé), le domaine de la saisie des données informatiques (anonymisation des données avant saisie, sécurisation de l'accès au fichier par mot de passe).

La diffusion ou la publication des résultats descriptifs de cette étude se fera dans un strict respect des mesures interdisant l'accès à des informations directement ou indirectement nominatives. L'Unité d'Epidémiologie de l'Institut Pasteur de Madagascar (IPM) sera responsable de l'archivage des questionnaires et les fiches d'examen sur support papier. Les données sur support informatique et sur support papier seront traitées dans le respect strict de la confidentialité. Leur accès sera sécurise: pièce spécifique et sécurisation des bases de données (accès par mot de passe). La base sera antonymie á la fin de l'étude.

# VII EXPLOITATION DES RESULTATS

L'analyse des données donnera lieu à un rapport écrit adressé au Ministère de la Santé et du Planning Familial de Madagascar.

Les communications écrites ou orales associeront tous les intervenants de l'étude, dans la liste des auteurs ou dans les remerciements, en fonction de leur contribution respective à l'étude et aux présentations effectuées.

# VIII FINANCEMENT

Cette étude sera financée par l'association « Médecins pour le Tiers Monde ».

# IX CALENDRIER

Avril 2011 Présentation du projet au comité d'éthique,

Préparation de la mission,

Recensement, randomisation, attribution traitement

Mai - Août 2011 Phase d'intervention

Septembre 2011 Extraction des puces restantes

Octobre 2011 Analyse des données

Novembre 2011- Février 2012 Rédaction du rapport de l'étude

Fin Février 2012 Première ébauche du manuscrit

Cette étude fera l'objet de sujet de thèse de doctorat en médecine d'une étudiante allemande, Marlene THIELECKE.

# X PARTICIPANTS

**1) Unité d'épidémiologie de l'Institut Pasteur de Madagascar**

**Dr Vincent RICHARD,** Docteur en médecine, chef de l'Unité d'épidémiologie de l'Institut Pasteur de Madagascar: responsable scientifique, responsable de l'analyse globale des données.

**Dr Vaomalala RAHARIMANGA,** Docteur en médecine, médecin de l'Unité d'épidémiologie de l'Institut Pasteur de Madagascar, coordinateur et investigateur de l'étude. - **Dr Charles Emile RAMAROKOTO,** Docteur en médecine, médecin de l'Unité d'épidémiologie de l'Institut Pasteur de Madagascar, investigateur de l'étude.

**Dr RAKOTOSON Lina**, Docteur en démographie de l'Unité d'épidémiologie de l'Institut Pasteur de Madagascar.

**2) Médecins s de l'Université Charité, Berlin**

**Prof Hermann FELDMEIER,** Docteur en médecine, Professeur de médecine tropicale à la faculté de médecine de Berlin: responsable scientifique, responsable de l'analyse globale des données.

**Marlene THIELECKE,** doctorante en médecine, Université Charité, Berlin: coordinateur et investigateur de l'étude.

**Dr Daniel PILGER,** Docteur en médecine, Université Charité, Berlin.

**3) Ministère de la Santé**

**Dr Fanomezantsoa Haja Lynah RANDRIAMANANTENA,** Docteur en médecine, Chef de service de Division de la lutte contre les Puces Chiques.

**Dr RAMIANDRISOA Erlie Daniel,** Docteur en médecine, Médecin Inspecteur Moramanga.

**Dr RAKOTONDRAMANANA,** Docteur en médecine, Médecin chef du Centre de Santé de Base Niveau II Andasibe: information de la communauté, prise en charge des pathologies dépistées lors de la visite des sujets et de l'extraction de puces chiques restantes après l'intervention.

# XI BIBLIOGRAPHIE

1. Eisele M, Heukelbach J, Van Marck E, Mehlhorn H, Meckes O, Franck S et al. Investigations on the biology, epidemiology, pathology and control *of T. penetrans* in Brazil: I. Natural history of puce chique in man. Parasitol Res 2003; 90:87-99.
2. De Oviedo GF. Historia general y natural de las Indias. Madrid: Ediciones Atlas, 1959.
3. Guerra F. Alexeio de Abreu [1568 - 1630], author of the earliest book on tropical medicine describing amoebiasis, malaria, typhoid fever, scurvy, yellow fever, dracontiasis, trichuriasis and puce chique in 1623. Journal of Tropical Medicine and Hygiene 1968; 71(3):55-69.
4. Staden von Homberg zu Hessen H. Wahrhaftige Historia und Beschreibung einer Landschaft der wilden, nacketen, grimmigen Menschenfresser Leuten, in der Neuen Welt America gelegen. Frankfurt am Main: Weigand Hahn, 1556.
5. de Lery J. Histoire d'un voyage fait en la terre du Brésil, autrement dite Amérique. Patmos Verlag Gmbh & Co. KG, 1578.
6. Hesse P. Die Ausbreitung des Sandflohs in Afrika. Geographische Zeitschrift (Hettner) 1899;522-530.
7. Henning G. Zur Geschichte des Sandflohs (*Sarcopsylla penetrans* L.) in Afrika. Naturwissenschaftliche Wochenschrift 1904; 20:310-312.
8. Hoeppli R. Early references to the occurrence of *Tunga penetrans* in Tropical Africa. Acta Tropica 1963; 20(2):143-152.
9. Ratovonjato J, Randriambelosoa J, Robert V. *Tunga penetrans* (Insecta, Siphonaptera, Tungidae) à Madagascar: une nuisance négligée. Revue Médicine Véterinaire 2008; 11:551-556.
10. Chadee DD. Distribution patterns of *Tunga penetrans* within a community in Trinidad, West Indies. J Trop Med Hyg 1994; 97:167-170.
11. Chadee DD. Puce chique among five communities in south-western Trinidad, West Indies. Ann Trop Med Parasitol 1998; 92(1):107-113.
12. Arene FOI. The prevalence of sand flea (*Tunga penetrans*) among primary and post-primary school pupils in Choba area of the Niger Delta. Public Health, London 1984; 98:282-283.
13. Chadee DD, Furlonge E, Naraynsingh C, Le Maitre A. Distribution and prevalence of *Tunga penetrans* in coastal south Trinidad, West Indies. Trans R Soc Trop Med Hyg 1991; 85:549.
14. Ade-Serrano MA, Chuks Ejezie G. Prevalence of puce chique in Oto-Ijanikin village, Badagry, Lagos State, Nigeria. Ann Trop Med Parasitol 1981; 75(4):471-472.
15. de Carvalho RW , De Almeida AB, Barbosa-Silva SC, Amorim M, Ribeiro PC, Serra-Freire N. The patterns of puce chique in Araruama Township, State of Rio de Janeiro, Brazil. Mem Inst Oswaldo Cruz 2003; 96:1-6.
16. Muehlen M, Heukelbach J, Wilcke T, Winter B, Mehlhorn H, Feldmeier H. Investigations on the biology, epidemiology, pathology and control of *Tunga penetrans* in Brazil: II. Prevalence, parasite load and topographic distribution of lesions in the population of a traditional fishing village. Parasitol Res 2003; 90:449­455.
17. Wilcke T, Harms G, Feldmeier H. Seasonal variation of puce chique in an endemic community. Am J Trop Med Hyg 2005; 72:145-149.
18. Joseph JK, Bazile J, Mutter J, Shin S, Ruddle A, Ivers L et al. Puce chique in rural Haiti: a community-based response. Transactions of the Royal Society of Tropical Medicine and Hygiene 2006; 100:970-974.
19. Ugbomoiko US, O foezie IE, Heukelbach J. Puce chique: high prevalence, parasite load, rural community in Lagos State, Nigeria. Int J Dermatol 2007; 46:475-481.
20. Heukelbach J, Jackson A, Ariza L, Lins Calheiros CM, de Lima Soares V, Feldmeier H. Epidemiology and clinical aspects of puce chique (sand flea infestation) in Alagoas State, Brazil. Journal of Infection in Developing Countries 2007; 1:202-209.
21. Blanchard RAE. Pr6sence de la chique (*Sarcopsylla penetrans*) à Madagascar. Archive de Parasitologie 1899; 2:627-630.
22. Rabary M. La chique à Madagascar. 1902.
23. Kehr JD, Heukelbach J, Mehlhorn H, Feldmeier H. Morbidity assessment in sand flea disease (puce chique). Parasitol Res 2007; 100:413-421.
24. Muehlen M, Feldmeier H, Wilcke T, Winter B. Identi fying risk factor for puce chique and heavy infestation in a resource-poor community in northeast Brazil. Trans Roy Soc Trop Med Hyg 2006; 100(4):371-380.
25. Ugbomoiko US, Ariza L, O foezie IE, Heukelbach J. Risk factors for puce chique in Nigeria: identi fication of targets for e ffective intervention. PloS Neglected Tropical Diseases 2007; Public Health (1):87.
26. Feldmeier H, Heukelbach J. Epidermal parasitic skin diseases: a neglected category of poverty-associated plagues. Bull Wrld Health Org 2007; in press: e324. doi:10.1371/journal.pntd.0000324.
27. Feldmeier H, Eisele M, Saboia Moura RC. Severe Puce chique in underprivileged communities: case series from Brazil. Emerging Infectious Diseases 2003; 9:949-955.
28. Ariza L, Seidenschwang M, Buckendahl J, Gomide M, Feldmeier H, Heukelbach J. Puce chique: a neglected disease causing severe morbidity in a shantytown in Fortaleza, State of Ceará. Rev Soc Bras Med Trop 2007; 40(1):63-67.
29. Wilcke T, Heukelbach J, Moura RSC, Kerr-Pontes LRS, Feldmeier H. High prevalence of puce chique in a poor neighbourhood in Fortaleza, Northeast Brazil. Acta Tropica 2002; 83:255-258.
30. Rietschel W. Beobachtungen zum Sandfloh (*Tunga penetrans*) bei Mensch und Hund in Französisch-Guayana. Tierärztliche Praxis 1989; 17(2):189-193.
31. Costa AML, Wilcke T, Mencke N, Feldmeier H. The animal reservoir of *Tunga penetrans* in severely affected communities of north-east Brazil. Medical Veterinary Entomology 2004; 18:329-335.
32. Heukelbach J, Mencke N, Feldmeier H. Cutaneous larva migrans and puce chique: the challenge to control zoonotic ectoparasitoses associated with poverty. Trop Med Int Hlth 2002; 7:907-910.
33. Witt LH, Heuckelbach J, Schwal fenberg S, Ribeiro RA, Harms G, Feldmeier H. Infestation of wistar rats with *Tunga penetrans* in different microenvironments. American Journal of Tropical Medicine and Hygiene 2007; 76(4):666-668.
34. Linardi PM, Calheiros CML, Campelo-Junior EB, DuarteE.M., Heukelbach J, Feldmeier H. Occurrence of the o ff-host li fe stages of *Tunga penetrans* (Siphonaptera) in various environments in Brazil. Ann Trop Med Parasitol 2010; 104(4):337-345.
35. Feldmeier H, Kehr JD, Poggensee G. High exposure to *Tunga penetrans* (Linnaeus, 1785) correlates with intensity of infestation. Mem Inst Oswaldo Cruz 2006; 101:65­69.
36. Feldmeier H, Heukelbach J, Eisele M, Sousa AQ, Barbosa LM, Carvalho CB. Bacterial superinfection in human puce chique. Trop Med Int Hlth 2002; 7(7):559­564.
37. Bruce CO, Knigin TD, Yolles SF. A discussion of the chigoe (*Tunga penetrans)* based on experiences in British Guiana. Military Surgeon 1942; 82:446-452.
38. Gordon RM. The jigger flea. Lancet 1941; 2:47-49.
39. Guyon M. Note accompagnant la présentation d'un ouvrage intitule: Histoire naturelle et médicale de la Chique, *Rhynchoprion penetrans* (Oken). Cahiers de Recherche de l’Academic de Science (Paris) 1870; 70:785-792.
40. Melo CR, Melo IS. Linfedema ele fantöide verrucoso associado a infestaçao por *Tunga penetrans*. An Bras Dermatol 1989; 64(1):35-37.
41. Obarrio M, Ghilhe E. Neuritis ascendente provocada por *Tunga penetrans*. Semana Med 1947; 9:53-57.
42. Troussaent M. Accidents cutanes graves produits par le Sarcopsylla penetrans (puce chique) et leur ressemblance avec l'ulcère phagedenique des pays chauds. Archives de médecine et de pharmacie militaires 1902; 39:42-50.
43. Bonnet G. Memoire sur la puce pénétrante ou chique (*Pulex penetrans*). Archives de médecine navale 1867; 8:19-53;81-119;259-285.
44. Cotes EC. The jigger or chigo pest. The Indian Medical Gazette 1899; 4:160-163.
45. Waterton C. Wanderings in South America, the North-West of the United States and the Antilles, in the years 1812, 1816, 1820 and 1824 with original instructions for the perfect preservation of birds and for cabinets of natural history. London: Oxford University Press, 1973.
46. Jolly GG. An entomological episode of the east African campaign. Indian Medical Gazette 1926; 61:164-165.
47. Tonge BL. Tetanus from chigger flea sores. J Trop Pediatr 1989; 35:94.
48. Litvoc J, Leite RM, Katz G. Aspectos epidemiolögicos do tetano no estado do Sao Paulo (Brasil). Revista Instituto Medicina Tropical de Säo Paulo 1991; 33(6):477-484.
49. Soria MF, Capri JJ. Tetanos y "pique". La Prensa Medica Argentina 1953; 40(1):4-11.
50. Obengui. La PUCE CHIQUE et le tetanos au C.H.U. de Brazzaville. Dakar Médical 1989; 34(1-4):44-48.
51. Schwalfenberg S, Witt LH, Kehr JD, Feldmeier H. Prevention of puce chique using a biological repellent: a small case series. Ann Trop Med Parasitol 2004; 98:89-94.
52. Feldmeier H, Kehr JD, Heukelbach J. A plant-based repellent protects against *Tunga penetrans* infestation and sand flea disease. Acta Tropica 2006; 99:126-136.
53. Heukelbach J, Pilger D, Oliveira F, Khakban A, Ariza L, Feldmeier H. A highly efficacious pediculocide based on dimeticone: Randomized observer blinded comparative trial. BMC Infectious Diseases 2008; 8(115): doi:10.1186/1471-2334-8-115.
54. Nair B. Final report on the safety assessment of stearoxy dimethicone, dimethicone, methicone, amino bispropyl dimethicone, aminopropyl dimethicone, amodimethicone, amodimethicone hydroxystearate, behenoxy dimethicone, C24-28 alkyl methicone, C30-45 alkyl methicone, C30-45 alkyl dimethicone, cetearyl methicone, cetyl dimethicone, dimethoxysilyl ethylenediaminopropyl dimethicone, hexyl methicone, hydroxypropyldimethicone, stearamidopropyl dimethicone, stearyl dimethicone, stearyl methicone, and vinyldimethicone. Toxicol 2003; 22:11-35.
55. Feldmeier H. Dimeticon-Präparate gegen Kopflausbefall. Deutsche Apothekerzeitung 2009; 149(6):87-95.
56. Heukelbach J, Eisele M, Jackson A, Feldmeier H. Topical treatment of puce chique: a randomized, controlled trial. Ann Trop Med Parasitol 2003; 97:743-749.
57. Heukelbach J, Winter B, Wilcke T, Muehlen M, Albrecht S, Sales de Oliveira FA et al. Selective mass treatment with ivermectin to control intestinal helminthiases and parasitic skin disease in a severely affected population. Bull Wrld Health Org 2004; 82:563-571.
58. Heukelbach J, Franck S, Feldmeier H. Therapy of puce chique: a double-binded randomized controlled trial with oral ivermectin. Mem Inst Oswaldo Cruz 2005; 99(8):873-876.
59. Chaccour C, Lines J, Whitty CJ. Effect of ivermectin on Anopheles gambiae mosqitoes fed on humans: the potential of oral insecticides in malaria control. Journal of Infectious Diseases 2010; 202(1):113-116.
60. Dunne CL, Malone CJ, Whitworth JAG. A field study of the effects of ivermectin on ectoparasites of man. Trans R Soc Trop Med Hyg 1991; 85:550-551.
61. Youssef YM, Sadaka HAH, Eissa MM, El-Ariny AF. Topical application of ivermectin for human ectoparasites. American Journal of Tropical Medicine and Hygiene 1995; 53(6):652-653.
62. Ariza L, Wilcke T, Jackson A, Gomide M, Ugbomoiko US, Feldmeier H et al. A simple method for rapid community assessment of puce chique. Trop Med Int Hlth 2010; 15(7):856-864.

# ANNEXE 1 : Classification de Fortaleza

La classification des puces chiques incorporées sera effectuée selon la méthode de Fortaleza (1) qui comporte 5 stades :

**Stade I** point noir (puce chique en voie de pénétration)

**Stade II** tâche blanche aplatie centrée par un point noir, prurigineuse avec un diamètre

de 1 à 2 millimètres

**Stade III** tâche blanche surélevée, plus ou moins douloureuse, en forme de pièce de

Monnaie d'un diamètre de 3 à 10 millimètres centrée par un point noir

**Stade IV** croûte noire formée par un tissu nécrotique. Le parasite mourant ou mort ne

sera pas différencié

**Stade V** résidu cicatriciel annulaire (l'épiderme a été débarrassé du reste du parasite)

Les lésions manipulées par le patient seront également documentées.

**Prurit:**

0 1 2 3 4


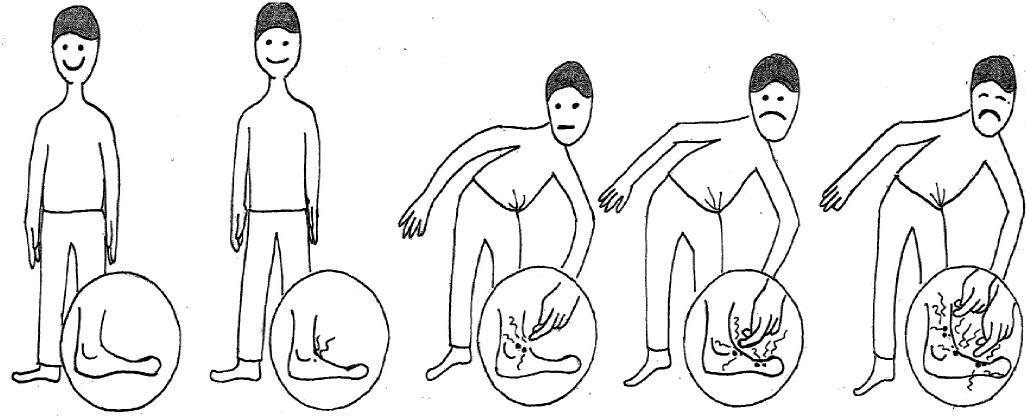


**Prurit nocturne:**

0 1 2 3 4


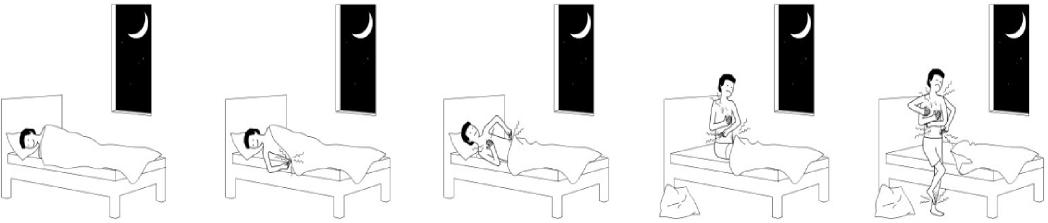


**Douleur spontane:**

0 1 2 3 4


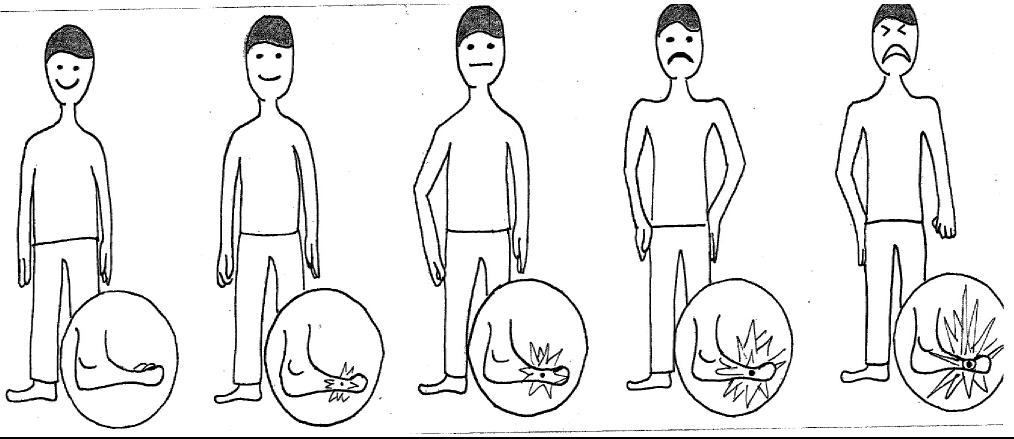


**Douleur nocturne:**

0 1 2 3 4


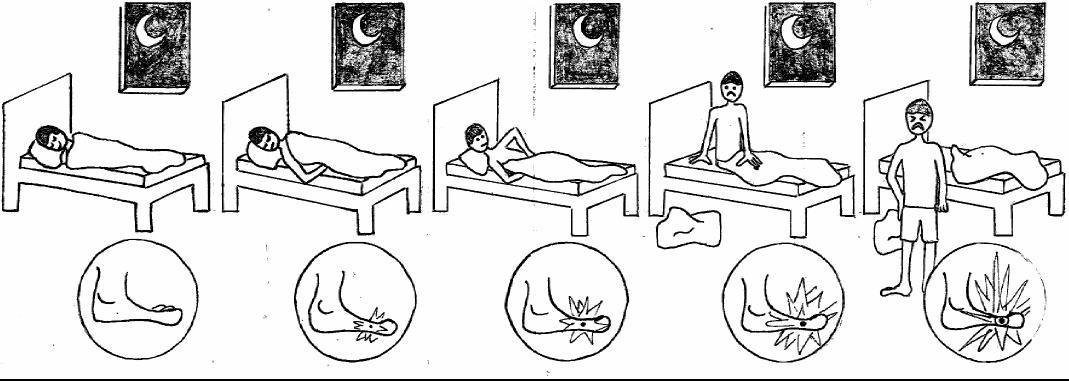


| 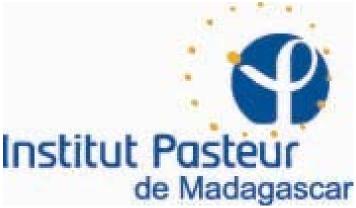 | Antananarivo, le 15 avril 2011.  Le Docteur **Vincent RICHARD** Chef de l'Unité Epidémiologie  A |
| --- | --- |

**Monsieur le Président**

du Comité d'Ethique auprès du Ministère de la Santé Publique

Monsieur le Président,

J'ai l'honneur de soumettre au Comité National d'Ethique auprès de Monsieur le Ministre de la Santé Publique et du Planning Familial le projet d'étude intitulé « PREVENTION DE LA PUCE CHIQUE ET DE LA MORBIDITE ASSOCIEE DANS UNE COMMUNAUTE A FAIBLE RESSOURCE A MADAGASCAR».

Dans le cadre de la réalisation de ce projet, je m'engage en tant que responsable scientifique de l'étude à respecter et à faire respecter par l'ensemble des investigateurs du projet, les principes éthiques retenus dans le cadre de la recherche biomédicale à Madagascar.

Je m'engage par ailleurs à fournir une copie de tous les rapports techniques élaborés dans le cadre de cette étude et à accéder à toute demande complémentaire du Comité National d'Ethique.

Je vous prie d'agréer, Monsieur le Président, l'assurance de ma très haute considération.

Le chef de l'Unité ou le responsable hiérarchique
